# Fifteen Years of Sm-p80-Based Vaccine Trials in Nonhuman Primates: Antibodies From Vaccinated Baboons Confer Protection *in vivo* and *in vitro* From *Schistosoma mansoni* and Identification of Putative Correlative Markers of Protection

**DOI:** 10.3389/fimmu.2020.01246

**Published:** 2020-06-19

**Authors:** Weidong Zhang, Loc Le, Gul Ahmad, Adebayo J. Molehin, Arif J. Siddiqui, Workineh Torben, Souvik Karmakar, Juan U. Rojo, Souad Sennoune, Samara Lazarus, Sabiha Khatoon, Jasmin Freeborn, Justin Sudduth, Ashraf F. Rezk, David Carey, Roman F. Wolf, James F. Papin, Ray Damian, Sean A. Gray, Florian Marks, Darrick Carter, Afzal A. Siddiqui

**Affiliations:** ^1^Center for Tropical Medicine and Infectious Diseases, Texas Tech University Health Sciences Center, Lubbock, TX, United States; ^2^Department of Internal Medicine, School of Medicine, Texas Tech University Health Sciences Center, Lubbock, TX, United States; ^3^Department of Natural Sciences, Peru State College, Peru, NE, United States; ^4^Department of Biology, University of Hail, Hail, Saudi Arabia; ^5^Department of Biological Sciences, Louisiana State University of Alexandria, Alexandria, LA, United States; ^6^Center for Cancer Research, National Cancer Institute, National Institutes of Health, Bethesda, MD, United States; ^7^Department of Molecular, Cellular and Biomedical Sciences, University of New Hampshire, Durham, NH, United States; ^8^Department of Pathology, University of Oklahoma Health Sciences Center, Oklahoma City, OK, United States; ^9^Oklahoma City VA Health Care System, Oklahoma City, OK, United States; ^10^Department of Cellular Biology, University of Georgia, Athens, GA, United States; ^11^PAI Life Sciences, Seattle, WA, United States; ^12^International Vaccine Institute, SNU Research Park, Seoul, South Korea; ^13^Department of Medicine, University of Cambridge, Cambridge, United Kingdom; ^14^Infectious Disease Research Institute, Seattle, WA, United States

**Keywords:** *Schistosoma mansoni*, schistosomiasis, baboons, Sm-p80 vaccine, systems biology, transcriptomics, passive transfer

## Abstract

Recent advances in systems biology have shifted vaccine development from a largely trial-and-error approach to an approach that promote rational design through the search for immune signatures and predictive correlates of protection. These advances will doubtlessly accelerate the development of a vaccine for schistosomiasis, a neglected tropical disease that currently affects over 250 million people. For over 15 years and with contributions of over 120 people, we have endeavored to test and optimize Sm-p80-based vaccines in the non-human primate model of schistosomiasis. Using RNA-sequencing on eight different Sm-p80-based vaccine strategies, we sought to elucidate immune signatures correlated with experimental protective efficacy. Furthermore, we aimed to explore the role of antibodies through *in vivo* passive transfer of IgG obtained from immunized baboons and *in vitro* killing of schistosomula using Sm-p80-specific antibodies. We report that passive transfer of IgG from Sm-p80-immunized baboons led to significant worm burden reduction, egg reduction in liver, and reduced egg hatching percentages from tissues in mice compared to controls. In addition, we observed that sera from Sm-p80-immunized baboons were able to kill a significant percent of schistosomula and that this effect was complement-dependent. While we did not find a universal signature of immunity, the large datasets generated by this study will serve as a substantial resource for further efforts to develop vaccine or therapeutics for schistosomiasis.

## Introduction

Schistosomiasis is a debilitating neglected tropical disease caused by infection with parasitic trematodes of the genus *Schistosoma*. Six species of *Schistosoma* cause clinical disease in humans, altogether responsible for over 290,000 deaths annually (1). While the rate of mortality is relatively low considering over 250 million people live with this disease (2), the clinical manifestations of schistosomiasis are chronic and insidious, including anemia, growth retardation, fever, genital lesions, hepatosplenomegaly and slow, irreversible organ damage (3, 4). These sequelae result in 3.31 million disability-adjusted life years (DALYS) lost according to recent estimates (5). Currently, schistosomiasis is endemic in 78 countries with over 800 million people at risk for infection (6).

For a myriad of reasons, control and elimination of schistosomiasis have eluded the research community and policy makers alike. While some success in reducing the spread of this disease have been achieved through integrated approaches combining mass drug administration (MDA), molluscicides, health education, behavior modification, and public works programs such as construction of concrete irrigation canals, schistosomiasis continues to be a major source of global health burden (7–9). Implementation of these integrated interventions can be logistical questions in economically strained communities such as rural villages in sub-Saharan Africa and southeast Asia (10, 11). It is within these communities, especially in high transmission hotspots, that MDA alone cannot result in the elimination of schistosomiasis as a public health concern (12). While mathematical modeling on the effectiveness of praziquantel (PZQ), the drug of choice used for antischistosome MDA, predicts against the emergence of drug resistance in the near future, overreliance and widespread repeated administration of PZQ may result in that future sooner rather than later (13, 14). Additionally, PZQ is not effective against juvenile schistosome parasites and does not prevent re-infection, necessitating repeated rounds of MDA for schistosomiasis control and elimination initiatives. Lapses in MDA can lead to rapid rebound of community infection rates to pre-treatment levels (15, 16). Hence, development of an antischistosome vaccine would be beneficial to achieve schistosomiasis elimination goals (17–19).

Sm-p80 is the large subunit of a schistosome calcium-activated neutral protease calpain (20), and has been tested for its vaccine efficacy in different vaccine strategies and formulations since 1997 (21). Although Sm-p80-based vaccines have been demonstrated to have many beneficial effects such as prophylactic (22) and therapeutic efficacy (23), cross-species protection against *S. haematobium* (24) and *S. japonicum* (25), immune correlates and mechanisms of protection against schistosomiasis remain poorly understood. While much has been learned from conventional immunological methods such as ELISA, Western blotting, ELISPOT, and even flow cytometry, recent development in systems biology and high throughput “omics” technologies have invited large paradigm shifts to vaccinology (26, 27). Using next-generation RNA sequencing (RNA-Seq), our group has reported some key molecular gene interactions associated with Sm-p80-based vaccine immunogenicity and efficacy (28, 29) as well as system-wide molecular interactions associated with trickle schistosome infections, chronic disease and PZQ treatment in the nonhuman primate model (29). In the present study, we aimed to explore immune signatures of Sm-p80-based vaccines through transcriptomic analyses of eight different strategies utilized across 15 years of preclinical studies using the baboon model, correlating with previously published efficacy results. Furthermore, we assessed the role of antibodies through *in vivo* passive transfer of IgG obtained from immunized baboons and *in vitro* killing of schistosomula using Sm-p80-specific antibodies.

## Materials and Methods

### Statement of Ethics

All animal procedures were conducted in accordance with Institutional Animal Care and Use Committee (IACUC) Guidelines (Protocol Number 20010202) and were approved by the Animal Ethics Committee at the Texas Tech University Health Sciences Center.

### Animals and Parasites

Female C57BL/6 mice (6–8 weeks old) were purchased from Charles River Laboratories (Wilmington, MA, USA). *Schistosoma mansoni* (Puerto Rico PR-1 strain)-infected *Biomphalaria glabrata* snails were procured from the Schistosome Resources Center (Biomedical Research Institute, Rockville, MD, USA).

### Source of Sera, IgG, and Cells

The sera used for heterologous passive transfer experiments were obtained from pooled sera of baboons immunized with Sm-p80-based vaccines and their control counterparts from eight previous studies. First, Sm-p80-VR1020 was a DNA vaccine formulation which elicited a robust antibody response and Th1-related cytokines such as interferon-gamma (IFNγ) (22). The second and third Sm-p80-based vaccine strategies used in this study were DNA prime with Sm-p80-VR1020 and protein boost with recombinant Sm-p80 and CpG ODN10104 (TLR9 agonist) or Resiquimod (TLR7 agonist) (30), yielding a nuanced and balanced pro-inflammatory and anti-inflammatory response as well as high IgG titers. The fourth and fifth strategies from which samples were obtained in this study were recombinant Sm-p80 formulated with CpG ODN10104 (TLR9 agonist) or Resiquimod (30), producing a similar immune response profile yet resulting in higher protection compared to the prime-boost counterparts. Finally, the sixth, seventh, and eighth vaccine strategies were recombinant Sm-p80 formulated with glucopyranosyl lipid adjuvant (GLA) as an aqueous formulation (AF), formulated with aluminum hydroxide (Alum), or formulated in a soluble oil-in-water emulsion (SE). Recombinant Sm-p80 formulated with GLA-SE (31) demonstrated the highest levels of protection while GLA-AF (unpublished data) and GLA-Alum (32) did not surpass previously tested strategies. These eight vaccination strategies followed a standard immunization schedule with 3-4 injections at 4-week intervals followed by parasite challenge with 1000 *S. mansoni* cercariae 4 weeks after the final injection. Eight weeks after parasite challenge, animals were sacrificed and adult worms were perfused from the portal system and mesenteric veins. Blood was collected after vaccination (to determine the effect of the vaccine), and 8 weeks after parasite challenge (to study vaccine-induced immune responses with schistosomiasis disease progression). Blood was also collected from animals in their respective control groups which received adjuvant control injections in place of the vaccine. Spleen and mesenteric lymph nodes samples were collected at necropsy.

IgG was purified from baboon sera pooled from all 8 aforementioned strategies using Pierce^TM^ Protein A/G Agarose kit (Thermo Fisher Scientific) according to manufacturer's protocol. IgG purity was analyzed by SDS-PAGE and concentration was measured by NanoDrop 1000 Spectrophotometer (Thermo Fisher Scientific). Peripheral blood mononuclear cells (PBMCs) from two time points, spleen cells, and mesenteric lymph nodes from the eight Sm-p80-based vaccine strategies and their respective controls were previously stored in freezing media (10% DMSO in fetal bovine sera and RPMI) at −80°C and were used for transcriptomic analyses *via* RNA-seq.

### Heterologous Passive Transfer and Parasite Challenge

Twenty C57BL/6 mice were randomly divided into control (*n* = 10) and experimental (*n* = 10) groups. Each mouse from the control group received an intravenous injection of purified IgG (75 μg) from control baboon sera while those in the experimental group received purified IgG (75 μg) from Sm-p80-immunized baboon sera at days 0, 3, and 9. At day 6, each mouse was challenged with 150 *Schistosoma mansoni* cercariae *via* tail immersion in water to allow for percutaneous infection. Two of the control mice died over the course of this study due to unknown reasons and were excluded from analysis.

### Determination of Worm Burden, Egg Burden, and Tissue Egg Hatching

Mice were euthanized six weeks post-cercarial challenge and adult worms were recovered from the mesenteric vasculature and hepatic portal system by perfusion (33). The percent reduction in adult worm burden was determined by comparing the number of worms recovered from the experimental groups (*I*) to the control group (*C*). Protection (*P*) was calculated using the formula: %*P* = [(*C* – *I*)/*C* × 100]. To assess the schistosome egg burden in tissues, livers and intestines of the euthanized mice were digested in 4% potassium hydroxide at 37°C overnight without CO_2_, washed, and resuspended in a 1.2% (w/v) sodium chloride solution (33). Eggs were enumerated under light microscopy to determine the number of eggs per gram in liver and intestine.

Tissue egg hatching rates were determined as previously described (29). Briefly, 0.5–1 g sections of liver and intestine were collected from each mouse following euthanasia. Tissue samples were placed in a solution of cold 1.2% NaCl and gently homogenized with a blender for 30 s. The homogenized samples were then passed through a series of sieves (180, 106, and 45 μm mesh opening). *Schistosoma mansoni* eggs were collected from the 45 μm sieve and centrifuged at 300 × *g* for 10 min. The egg pellet was resuspended in fresh water after discarding the supernatant and seeded into 24-well plates. These plates were placed under a light source for 2 h to induce egg hatching. Mature eggs and hatched eggs were counted using a light microscope and the egg hatching rates were expressed as the percentage of eggs hatched compared to mature eggs.

### *In vitro* Killing of *Schistosoma mansoni* Schistosomula

Sera for *in vitro* schistosomula killing assay were obtained as described above. *Schistosoma mansoni* cercariae collected from infected snails were mechanically transformed into schistosomula by repeated transfers through a 22 gauge syringe needle (33). Approximately 50 schistosomula were seeded into each well of a 24-well plate and cultured overnight in complete media (RPMI 1640 supplemented with 10% fetal bovine serum, 100 μg/mL penicillin G, 100 μg/mL streptomycin and 10 μg/mL gentamycin) in the presence or absence of Sm-p80-immunized baboon sera (1:50 dilution) with or without exogenous complement sera from guinea pig (1:50 dilution) (Millipore Sigma). Media alone or pooled sera from baboons receiving adjuvant/vehicle alone were used as controls. Sera were heat-inactivated by incubating samples at 56°C for 30 min. Schistosomula were classified as viable or dead on the basis of motility and morphology (presence of granularity and blebbing) under light microscopy with 3 technical replicates per group.

### RNA Purification and RNA-Sequencing

Total RNA was used for RNA-seq library preparation from individual baboon samples of PBMCs, spleen cells, and lymph node cells with three biological replicates and two technical replicates along with their corresponding controls for each of the eight vaccine strategies. Total RNA was extracted using GenElute® Mammalian Total RNA Miniprep Kit (Sigma–Aldrich, St. Louis, MO) following the manufacturer's instructions. Total RNA concentrations were quantified via Qubit® 3.0 Fluorometer and RNA HS Assay Kit (Thermo Fisher,Waltham, MA) and RNA quality was determined using the Agilent 2200 TapStation (Agilent, Santa Clara, CA).

Library preparation was completed as previously described (31) and stranded mRNA-sequencing on the NovaSeq 6000 platform (Illumina) was fulfilled following standard protocols by the Texas Tech University Center for Biotechnology and Genomics (Lubbock, TX).

### RNA-Seq Analysis Pipeline

Alignment and determination of differentially expressed genes (DEGs) were conducted using the Galaxy platform (www.usegalaxy.org). Briefly, raw reads were aligned to the human genome (hg19) using HISAT2 (34). Then, read summarization was completed using the featureCounts program (35). DEGs were determined from the resultant count tables using DESeq2 (36).

Downstream pathway analyses on DEGs were completed based on cut-offs of 1.5-fold change compared to control groups and *P* < 0.1. Gene ontology (GO) of both up- and down-regulated DEGs were analyzed using ClueGo (version 2.5.6) app on Cytoscape (version 3.7.2) software. DEGs related to immune system processes were visualized as a network of GO term clusters with a minimum of 3 genes per cluster unless otherwise mentioned, represented likewise as a pie graph depicting percentage of genes per GO group out of the total number of genes. Pathway analyses were generated with Ingenuity Pathway Analysis (IPA) (Qiagen, Venlo, Netherlands) using genes related to immune system processes. Heat maps depicting gene expression and bar graphs representing Z-scores and *P*-values for canonical pathways were generated using GraphPad Prism (version 7.04).

Selected genes validated via quantitative real-time polymerase chain reaction (qPCR) as previously described (31). Briefly, primers were designed from mRNA sequences obtained from the NCBI for *Papio anubis* genes. The list of primer sequences used for qRT-PCR is provided in Supplementary Table 1. Total RNA from PBMCs, spleen cells, and lymph node cells was extracted using GenElute™ Mammalian Miniprep kit (Sigma-Aldrich, St. Louis, MO, USA) and first strand cDNA synthesis was completed using the Maxima First Strand cDNA synthesis kit (Thermo Fisher Scientific). Amplification of selected genes was carried out using SYBR Premix Ex Taq™ (TIi RNase H Plus; Takara, Japan) in triplicates on a StepOne™ plus Real-time PCR system (Thermo Fisher Scientific). Results were analyzed using DataAssist™ software (version 3.0) (Thermo Fisher Scientific) (Supplementary Table 2).

### Statistical Analysis

Student's *t*-tests were used for pairwise comparisons between groups for the mouse *in vivo* passive transfer and *in vitro* schistosomula killing studies with *P* < 0.05 indicating statistical significance. Plotted data represents individual data points with error bars showing means and standard error of the mean. Differentially expressed genes were selected based on the cutoff of *P* < 0.1 and 1.5-fold change compared to controls for pathway analyses using IPA and ClueGO.

## Results

### Reduction in Parasitological Burden Following Passive Transfer

**Figure 1:**
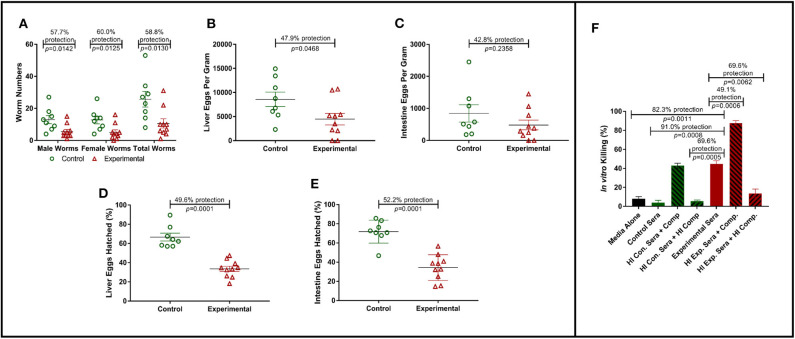
Role of antibodies in Sm-p80-based vaccine-mediated protection. **(A)** Recovered adult male and female worms following passive transfer of purified IgG from baboons immunized with Sm-p80-based vaccine. **(B)** Liver egg burden and **(C)** intestine egg burden at necropsy. Hatching rates of eggs recovered from livers **(D)** and Intestines **(E)** of mice at necropsy. **(F)** *In vitro* killing of *S. mansoni* schistosomula in the presence of pooled sera from Sm-p80-vaccinated baboons with addition or absence of exogenous complement. All mice were sacrificed at 6 weeks post *S. mansoni* cercariae challenge. Plotted data represents individual data points with error bars showing means and standard error of the mean.

To assess the protective efficacy of Sm-p80-specific IgG against *S. mansoni* infections, mice were passively transferred with purified Sm-p80-specific IgG from pooled baboon sera as described above. Passively transferred Sm-p80-specific IgG protected naïve C56BL/6 mice from *S. mansoni* challenge infection. Specifically, we observed significant reduction in adult worm numbers [male worms: 57.7% (*P* = 0.0142); female worms: 60.0% (*P* = 0.0125); total worms: 58.8% (*P* = 0.0130)] in experimental mice that received Sm-p80-specific antibodies when compared to the control mice (Figure 1A). Liver and intestine tissue samples from control and experimental groups were also assessed for tissue egg burden. We observed a significant reduction of 47.9% (*P* = 0.0468) in liver egg burden in experimental mice when compared to the control group (Figure 1B). While we also observed a 42.8% (*P* = 0.2358) reduction in egg burden within the intestines of the experimental mice, the reduction was not statistically significant (Figure 1C).

### Reduction in *Schistosoma mansoni* Egg Viability in Mouse Tissues

In order to determine egg viability, we compared the hatching rates of eggs isolated from infected mouse liver and intestine tissue samples from the control and experimental groups. Hatching rate analysis of eggs isolated from the tissues of mice receiving Sm-p80-specific IgG showed a significant reduction of 52.2% (*P* = 0.0001) in the liver when compared to that of the control animals (Figure 1D). Similarly, we observed a significant decrease of 49.6% (*P* = 0.0001) in egg hatching rates from eggs isolated from the intestines of mice from experimental group compared to those from the control group (Figure 1E).

### *In vitro* Killing of Schistosoma *Schistosoma mansoni*

*Schistosoma mansoni* schistosomula were cultured in the presence of baboon sera with or without exogenous complement. In the presence of Sm-p80-immunized baboon sera, we observed a 91.0% killing efficiency (*P* = 0.0008) of schistosomula *in vitro* when compared to control sera. The killing efficacy was significantly augmented (1.7-fold, *P* = 0.0006) with the addition of exogenous complement compared to experimental sera without modification. The complement-dependent killing effect was reversed by heat-inactivation (Figure 1F). Interestingly, we also observed that the control sera with exogenous complement showed schistosomula killing efficiency comparable to the experimental sera alone.

### Immune Signatures of Sm-p80-Based DNA Vaccine

RNA-Seq analysis of gene expression in the PBMCs, spleen cells, and lymph nodes of baboons vaccinated with different strategies of Sm-p80-based vaccines revealed diverse immune signatures depending on the strategy and adjuvant choice. We performed gene ontology (GO) enrichment analysis to first determine which DEGs were related to immune system processes. GO terms reveal the processes to which the DEGs belong and the connection of processes which are represented by linked clusters of GO terms (Supplementary Figures 1–32).

**Figure 2:**
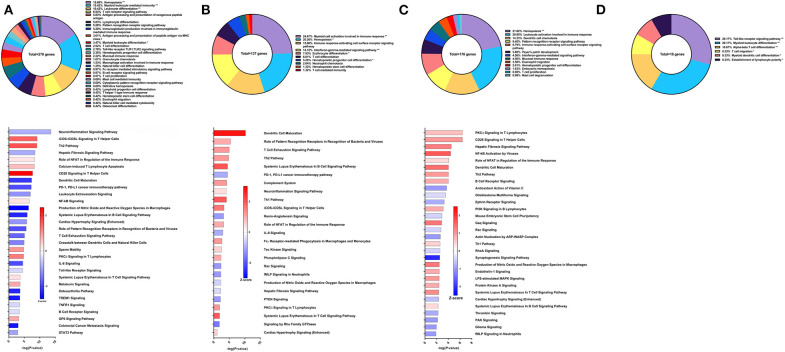
RNA-Seq analysis of Sm-p80-VR1020, a DNA vaccine strategy. **(A)** PBMCs after vaccination (Top) Gene ontology enrichment analysis represented as a pie graph of percentages of genes per group out of a total number of differentially expressed genes. “**” and “*” indicates *P* < 0.05 and *P* < 0.01, respectively. (Bottom) Canonical pathway analysis generated using IPA. Bars are plotted based on the – log10(*P*-value) and colored based on predicted activation (red) and deactivation/inhibition (blue) according to the Z-score, a composite assessment based on the degree of overlap between directional expression of genes from the observed data and the Qiagen-curated public database. The top 30 pathways are shown based on the lowest *P*-values. **(B)** PBMCs after challenge (Top) Gene ontology enrichment analysis. (Bottom) Canonical pathway analysis generated using IPA. **(C)** Spleen cells (Top) Gene ontology enrichment analysis. (Bottom) Canonical pathway analysis generated using IPA. **(D)** Mesenteric lymph node cells (Top) Gene ontology enrichment analysis. Bottom) Canonical pathway analysis generated using IPA.

Sm-p80-VR1020 was a DNA vaccine formulation which conferred 46% reduction in worm burden in baboons compared to controls (22). Four weeks after vaccination and prior to challenge, 270 genes related to immune system processes were differentially expressed in the PBMCs. These genes belonged to GO groups such as hemopoiesis (15.69%), leukocyte differentiation (10.42%), immunoglobulin production involved in immunoglobulin mediated immune response (5.28%), and others (Figure 2A top). These DEGs were inputted into IPA to predict activation (positive) or deactivation (negative) based on the *Z*-score, a composite of the degree of overlap between directional expression of genes from the observed data and the QIAGEN-curated public database. The pathways at this time point with the highest statistical significance include the neuroinflamation signaling pathway (Z-score = −0.853), iCOS-iCOSL signaling in T helper cells (Z-score = 1.508), and Th2 pathway (Z-score = 1.667) (Figure 2A bottom). Interestingly, the hepatic fibrosis signaling pathway was predicted to be deactivated (Z-score = −0.894) prior to parasite challenge. Predicted activation of the CD28 signaling in T helper cells pathway (Z-score = 2.449) and iCOS-iCOSL signaling in T helper cells pathway may explain why DEGs belonging to the immunoglobulin production involved in immunoglobulin mediated immune response GO group were the third highest group at 5.28% out of a total of 270 genes. High Sm-p80-specific IgG titers were previously correlated to protect baboons with this DNA vaccine strategy (22).

After schistosome parasite challenge and prior to sacrifice, blood was drawn and PBMCs were isolated for RNA-Seq analysis on baboons vaccinated with the DNA vaccine Sm-p80-VR1020 (Figure 2B top). At this time point, myeloid cell activation involved in immune response (24.67%), immune response-activating cell surface receptor signaling pathway (15.86%), and IFNγ-mediated signaling pathway (14.1%) were the top GO groups represented by the 137 DEGs (Figure 2B top). IPA predicted activation of pathways including dendritic cell maturation (Z-score = 2.309), role of pattern recognition receptor (PRR) recognition of bacteria and viruses (Z-score = 0.816), T cell exhaustion signaling pathway (Z-score = 1), and the Th2 pathway (Z-score = 1) (Figure 2B bottom). IPA also predicted involvement of the complement system (Z-score = 1) as well as the Th1 pathway (Z-score = 1.633).

At sacrifice, spleen and lymph node cells were collected for RNA-Seq analysis. The top GO groups for spleen cells include hemopoiesis (21.82%), leukocyte activation involved in immune response (20.85%), and dendritic cell chemotaxis (14.33%) (Figure 2C top). From these immune system process-associated DEGs, the canonical pathways predicted with most the statistical significance include PKCθ signaling in T lymphocytes (Z-score = 0.632), CD28 signaling in T helper cells (Z-score = 1), hepatic fibrosis signaling pathway (Z-score = 1.265), NF-kB activation by viruses (Z-score = 1.633), and role of NFAT in regulation of the immune response (Z-score = 0.378) (Figure 2C bottom). Interestingly, both Th1 and Th2 pathways were predicted to be activated in the spleen. RNA-Seq analysis of lymph nodes revealed 18 immune system process-related DEGs with no predicted canonical pathways that were statistically significant (Figure 2D).

### Immune Signatures of Sm-p80-Based DNA Prime/Protein Boost Vaccine With CpG ODN

**Figure 3:**
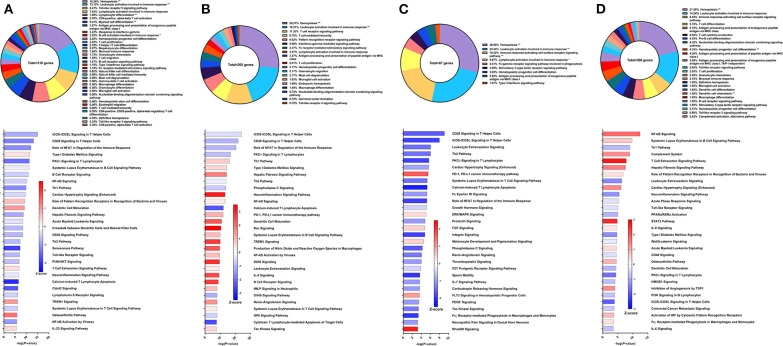
RNA-Seq analysis of Sm-p80-VR1020 + rSm-p80+ODN10104, a prime/boost vaccine strategy. **(A)** PBMCs after vaccination (Top) Gene ontology enrichment analysis represented as a pie graph of percentages of genes per group out of a total number of differentially expressed genes. “**” and “*” indicates *P* < 0.05 and *P* < 0.01, respectively. (Bottom) Canonical pathway analysis generated using IPA. Bars are plotted based on the – log10(*P*-value) and colored based on predicted activation (red) and deactivation/inhibition (blue) according to the Z-score, a composite assessment based on the degree of overlap between directional expression of genes from the observed data and the Qiagen-curated public database. The top 30 pathways are shown based on the lowest *P*-values. **(B)** PBMCs after challenge (Top) Gene ontology enrichment analysis. (Bottom) Canonical pathway analysis generated using IPA. **(C)** Spleen cells (Top) Gene ontology enrichment analysis. (Bottom) Canonical pathway analysis generated using IPA. **(D)** Mesenteric lymph node cells (Top) Gene ontology enrichment analysis. (Bottom) Canonical pathway analysis generated using IPA.

The second Sm-p80-based vaccine strategy used in this study was DNA prime with Sm-p80-VR1020 and protein boost with recombinant Sm-p80 and CpG ODN10104, a TLR9 agonist which resulted in 47.34% reduction of adult worms in vaccinated baboons compared to controls. After vaccination and prior to parasite challenge, 316 DEGs related to immune system processes were detected in the PBMCs. The GO groups with the largest percentage of these genes were hemopoiesis (16.24%), leukocyte activation involved in immune response (13.13%), and TLR9 signaling pathway (8.41%) (Figure 3A top). Many of the top statistically significant canonical pathways were predicted to be deactivated, including iCOS-iCOSL signaling in T helper cells, CD28 signaling in T helper cells, and the role of NFAT in regulation of the immune response (Figure 3A bottom). At this time point, Th1 and Th2 pathways were predicted to be deactivated while the IL-23 signaling pathway, crucial to the Th17 pathway, was predicted to be activated.

Prior to sacrifice, we detected 205 DEGs in the PBMCs compared to control animals. The top GO groups at this time point include hemopoiesis (26.27%), leukocyte activation involved in immune response (18.31%), T cell receptor signaling pathway (11.33%), and T cell mediated immunity (6.75%) (Figure 3B top). While several of the top canonical pathways did not change direction, such as iCOS-iCOSL signaling in T helper cells, CD28 signaling in T helper cells, and the role of NFAT, the prediction for the Th1 pathway (Z-score = 0.728) and B cell receptor signaling (Z-score = 2.673) converted to activation (Figure 3B bottom). Production of nitric oxide and reactive oxygen species in macrophages and iNOS signaling became among the top 30 most significant canonical pathways and both were strongly predicted to be activated.

GO enrichment analysis of the spleen cells revealed similar representation of the top groups, including hemopoiesis (28.95%), leukocyte activation involved in immune response (24.34%), and immune response-activating cell surface receptor signaling pathway (18.42%) (Figure 3C top). Likewise, the top statistically significant pathways include CD28 signaling in T helper cells, iCOS-iCOSL signaling in T helper cells, leukocyte extravasation signaling, and the Th2 pathway, all of which were predicted to be deactivated (Figure 3C bottom). Gene expression from lymph node cells revealed different immune signatures compared to those found from PBMCs and spleen cells. Besides hemopoiesis, leukocyte activation involved in immune response, and immune response-activating cell surface receptor signaling pathway, GO enrichment analysis indicated T cell differentiation (5.7%), pro-B cell differentiation (4.35%) as among the highest represented GO groups out of 306 DEGs related to immune system processes (Figure 3D top). The DEGs in the lymph nodes also revealed the involvement of different canonical pathways compared to PBMCs and spleen cells in this strategy. For example, the complement system (Z-score = 1.342) and the hepatic fibrosis signaling pathway (Z-score = 1.528) were predicted to be activated.

### Immune Signatures of Sm-p80-Based DNA Prime/Protein Boost Vaccine With Resiquimod

**Figure 4:**
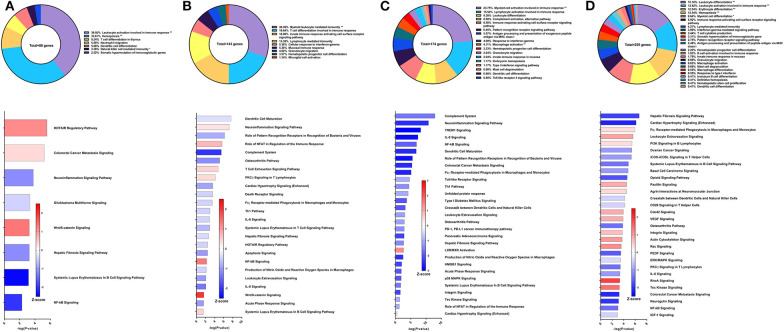
RNA-Seq analysis of Sm-p80-VR1020 + rSm-p80+Resiquimod, a prime/boost vaccine strategy. **(A)** PBMCs after vaccination (Top) Gene ontology enrichment analysis represented as a pie graph of percentages of genes per group out of a total number of differentially expressed genes. “**” and “*” indicates *P* < 0.05 and *P* < 0.01, respectively. (Bottom) Canonical pathway analysis generated using IPA. Bars are plotted based on the – log10(*P*-value) and colored based on predicted activation (red) and deactivation/inhibition (blue) according to the Z-score, a composite assessment based on the degree of overlap between directional expression of genes from the observed data and the Qiagen-curated public database. The top 30 pathways are shown based on the lowest *P*-values. **(B)** PBMCs after challenge (Top) Gene ontology enrichment analysis. (Bottom) Canonical pathway analysis generated using IPA. **(C)** Spleen cells (Top) Gene ontology enrichment analysis. (Bottom) Canonical pathway analysis generated using IPA. **(D)** Mesenteric lymph node cells (Top) Gene ontology enrichment analysis. (Bottom) Canonical pathway analysis generated using IPA.

Resiquimod used as the adjuvant component of the Sm-p80-based vaccine in a DNA prime/protein boost strategy resulted in 37.62% reduction in schistosome worm burden (30). The efficacy of this vaccine strategy was lower on the spectrum of Sm-p80-based vaccines and may explain the relatively low number of DEGs related to immune system processes from PBMCs after vaccination but prior to parasite challenge. The majority of these genes belong to the GO groups leukocyte activation involved in immune response (39.5%), hemopoiesis (33.61%), T cell differentiation in thymus (9.24%), and dendritic cell differentiation (5.88%) (Figure 4A top). Pathway analysis using IPA revealed relatively few significant pathways including HOTAIR regulatory pathway (Z-score = 1.134), colorectal cancer metastasis signaling (Z-score = 0.378), neuroinflammation signaling pathway (Z-score = −1.134), and glioblastoma multiforme signaling (Z-score = −0.447) (Figure 4A bottom). The DEGs driving the predictions for these pathways primarily include matrix metallopeptidases and Wnt family members.

After parasite challenge, the top GO groups in PBMCs include myeloid leukocyte mediated immunity (30.36%), T cell differentiation involved in immune response (19.64%), and innate immune response activating cell surface receptor signaling pathway (16.96%) (Figure 4B top). IPA analysis revealed that pathways related to PCKθ signaling including role of NFAT (Z-score = 0.707) and NF-kB signaling (Z-score = 1.342) were predicted to be deactivated (Figure 4B bottom). Interestingly, complement system was predicted to be deactivated (Z-score = −2), possibly correlating with the reduced worm burden reduction of this strategy compared to other Sm-p80 vaccine strategies.

RNA-Seq and GO enrichment analysis on spleen cells indicated that the top GO groups were myeloid cell activation involved in immune response (23.75%), lymphocyte activation involved in immune response (15.54%), leukocyte differentiation (9.38%) and complement activation, alternative pathway (8.5%) (Figure 4C top). Although we see that 8.5% of the 174 DEGs related to immune system process belong to the complement activation GO group, IPA analysis revealed that the complement system pathway was predicted to be deactivated (Z-score = −1.633) with the highest statistical significance compared to other canonical pathways. Other significant pathways predicted to be deactivated include neuroinflammation signaling pathway (Z-score = −2.5), TREM1 signaling (Z-score = −3), IL-8 signaling (Z-score = −2.887), and NF-kB signaling (Z-score = −1.897) (Figure 4C bottom). The top GO groups for lymph node cells were leukocyte differentiation (15.16%), leukocyte activation involved in immune response (12.82%), and hemopoiesis (12.54%) (Figure 4D top). Similar to the PBMCs and spleen cells, IPA predictions for PCKθ-related signaling were deactivation, including NF-kB signaling (Z-score = −1.414), CD28 signaling (Z-score = −0.447), and iCOS-iCOSL signaling (Z-score = −1) (Figure 4D bottom). Interestingly, the hepatic fibrosis signaling pathway was predicted to be deactivated at both time points for PBMCs and for spleen and lymph node cells with this vaccine strategy.

### Immune Signatures of Sm-p80-Based Protein Vaccine With CpG ODN

**Figure 5:**
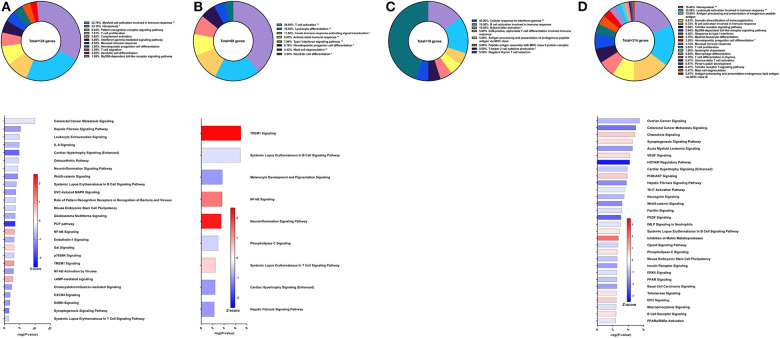
RNA-Seq analysis of rSm-p80+ODN10104, a recombinant protein vaccine strategy. **(A)** PBMCs after vaccination (Top) Gene ontology enrichment analysis represented as a pie graph of percentages of genes per group out of a total number of differentially expressed genes. “**” and “*” indicates *P* < 0.05 and *P* < 0.01, respectively. (Bottom) Canonical pathway analysis generated using IPA. Bars are plotted based on the – log10(*P*-value) and colored based on predicted activation (red) and deactivation/inhibition (blue) according to the Z-score, a composite assessment based on the degree of overlap between directional expression of genes from the observed data and the Qiagen-curated public database. The top 30 pathways are shown based on the lowest *P*-values. **(B)** PBMCs after challenge (Top) Gene ontology enrichment analysis. (Bottom) Canonical pathway analysis generated using IPA. **(C)** Spleen cells (Top) Gene ontology enrichment analysis. (Bottom) Canonical pathway analysis generated using IPA. **(D)** Mesenteric lymph node cells (Top) Gene ontology enrichment analysis. (Bottom) Canonical pathway analysis generated using IPA.

Compared to the DNA prime/protein boost strategy with CpG ODN, the recombinant protein vaccine strategy with CpG ODN conferred higher worm burden reduction at 57% compared to control animals (30). Using PBMCs obtained after vaccination, the top GO groups from which the immune system process-related DEGs belong include myeloid cell activation involved in immune response (32.78%), hemopoiesis (23.16%), pattern recognition receptor signaling pathway (9.04%), and T cell proliferation (7.91%) (Figure 5A top). Although the IFNγ-mediated signaling pathway was represented in the GO analysis through differential expression of IL-12 receptor subunit β1, there was insufficient data to predict activation or deactivation of the Th1 pathway. Similarly, Th2 and Th17 pathways were unable to be predicted (Figure 5A bottom). Instead, the top canonical pathways for PBMCs after vaccination and prior to parasite challenge were colorectal cancer metastasis signaling (Z-score = −0.277), hepatic fibrosis signaling pathway (Z-score = −1), leukocyte extravasation signaling (Z-score = −0.378), and IL-8 signaling (Z-score = −0.707), all of which were predicted to be deactivated.

From the 69 DEGs related to immune system processes identified in the PBMCs after parasite challenge, the top GO groups were T cell activation (38.95%), leukocyte differentiation (18.58%), innate immune response-activating signal transduction (11.5%), and antimicrobial humoral response (8.85%) (Figure 5B top). The top pathways that IPA predicted to be activating based on these DEGs include TREM1 signaling (Z-score = 2.449), NF-kB signaling (Z-score = 1.342), and neuroinflammation signaling pathway (Z-score = 2.236) driven by upregulation of DEGs such as TLR8, TLR9, TLR10, PLCG2, and others (Figure 5B bottom). The top pathways predicted to be deactivated were systemic lupus erythematosus in B cell signaling pathway (Z-score = −0.333), melanocyte development and pigmentation signaling (Z-score = −1), and phospholipase C signaling (Z-score = −0.447) mainly elicited by TNF family regulators, LAT, and PLCG2.

From the spleen, only 19 DEGs were related to immune system processes and these belong to GO groups such as cellular response to IFNγ (45.0%), natural killer cell activation (15.0%), and B cell response involved in immune response (15.0%). These few DEGs were insufficient to predict the direction of any canonical pathways (Figure 5C). The lymph nodes reveal much more about this vaccine strategy with 314 DEGs related to immune system processes. The majority of these DEGs belong to the following GO groups: hemopoiesis (18.44%), leukocyte activation involved in immune response (16.88%), antigen processing and presentation of exogenous peptide antigen (15%), and somatic diversification of immunoglobulins (8.91%) (Figure 5D top). The top canonical pathways predicted to be deactivated include ovarian cancer signaling (Z-score = −1), colorectal cancer metastasis signaling (Z-score = −1.941), and acute myeloid leukemia signaling (Z-score = −1.414), associated with Wnt family genes, RAS family genes, and SRC family kinases. Chemokine signaling (Z-score = 0.707), synaptogenesis signaling pathway (Z-score = 0.535), and VEGF signaling (Z-score = 0.447) were the top canonical pathways predicted to be activated by IPA (Figure 5D bottom).

### Immune Signatures of Sm-p80-Based Protein Vaccine With Resiquimod

**Figure 6:**
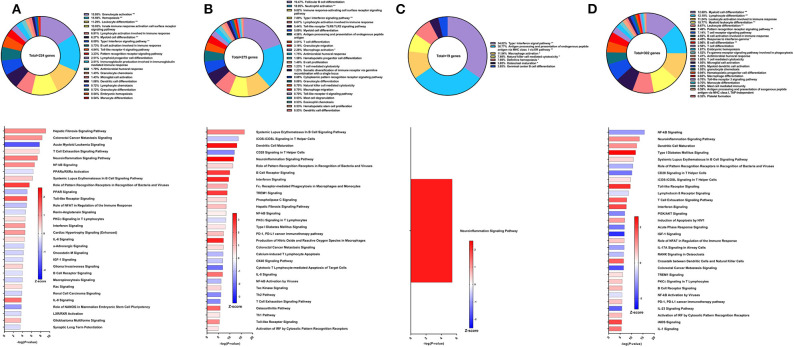
RNA-Seq analysis of rSm-p80+Resiquimod, a recombinant protein vaccine strategy. **(A)** PBMCs after vaccination (Top) Gene ontology enrichment analysis represented as a pie graph of percentages of genes per group out of a total number of differentially expressed genes. “**” and “*” indicates *P* < 0.05 and *P* < 0.01, respectively. (Bottom) Canonical pathway analysis generated using IPA. Bars are plotted based on the – log10(*P*-value) and colored based on predicted activation (red) and deactivation/inhibition (blue) according to the Z-score, a composite assessment based on the degree of overlap between directional expression of genes from the observed data and the Qiagen-curated public database. The top 30 pathways are shown based on the lowest *P*-values. **(B)** PBMCs after challenge (Top) Gene ontology enrichment analysis. (Bottom) Canonical pathway analysis generated using IPA. **(C)** Spleen cells (Top) Gene ontology enrichment analysis. (Bottom) Canonical pathway analysis generated using IPA. **(D)** Mesenteric lymph node cells (Top) Gene ontology enrichment analysis. (Bottom) Canonical pathway analysis generated using IPA.

The fifth strategy from which samples were obtained in this study was recombinant Sm-p80 formulated with Resiquimod (30), yielding higher reduction in worm burden than its prime/boost counterpart at 52.10% compared to controls in the baboon model. Using PBMCs obtained after vaccination and prior to parasite challenge, RNA-Seq analysis indicated 224 DEGs related to immune system processes. Most of these DEGs fall under GO groups such as granulocyte activation (15.95%), hemopoiesis (15.59%), leukocyte differentiation (11.29%), type I interferon signaling pathway (6.09%), and innate immune response activating cell surface receptor signaling pathway (10.93%) (Figure 6A top). Some of the canonical pathway with highest statistical significance were predicted to be in the opposite direction in this protein vaccine strategy compared to the prime/boost strategy with Resiquimod, including hepatic fibrosis signaling pathway (Z-score = 1.147), NF-kB signaling (Z-score = 1.155), and systemic lupus erythematosus in B cell signaling pathway (Z-score = 0.535). Interferon signaling (Z-score = 1), PKCθ signaling in T lymphocytes (Z-score = 0.333), Toll-like receptor signaling (Z-score = 1.633), IL-6 signaling (Z-score = 0.378), and IL-8 signaling (Z-score = 1.414) are other notable canonical pathways were predicted to be activated due to the effect of vaccination alone (Figure 6A bottom).

After parasite challenge, GO enrichment analysis of PBMCs indicated that DEGs fell into groups such as follicular B cell differentiation (19.47%), neutrophil activation (18.95%), immune response-activating cell surface receptor signaling pathway (9.82%), and type I interferon signaling pathway (7.89%) (Figure 6B top). IPA predictions for several canonical pathways did not differ in direction compared to the previous time point, including systemic lupus erythematosus in B cell signaling pathway (Z-score = 1.732), hepatic fibrosis signaling pathway (Z-score = 0.894), Toll-like receptor signaling (Z-score = 1.633), interferon signaling (Z-score = 3), and NF-kB signaling (Z-score = 0.258). Notably, the Th1 pathway (Z-score = 0.333) was predicted to be activated while the Th2 pathway (Z-score = −1.633) was predicted to be deactivated (Figure 6B bottom).

Unlike the prime/boost vaccine strategy using Resiquimod, the recombinant protein vaccine with Resiquimod had a minor effect on the transcriptome of spleen cells with only 19 DEGs identified. The largest GO groups for the spleen cell DEGs included type I interferon signaling pathway (34.62%), antigen processing and presentation of endogenous peptide antigen via MHC class I via ER pathway (30.7%), and macrophage activation (11.54%) (Figure 6C top). The only significant canonical pathway predicted by IPA was neuroinflammation signaling pathway (Z-score = 2.236) which was associated with upregulation in MHC I isotypes A and B, IFNγ receptor 2, TGFβ receptor 3, and VCAM1 (Figure 6C bottom). The response in the mesenteric lymph nodes was much more robust than the spleen with 302 DEGs related to immune response processes. The top GO groups representing these DEGs include myeloid cell differentiation (12.88%), lymphocyte differentiation (12.18%), leukocyte activation involved in immune response (11.24%), myeloid leukocyte differentiation (10.77%), and B cell activation involved in immune response (4.92%) (Figure 6D top). Unlike the predictions in the PBMCs after parasite challenge, NF-kB signaling in lymph node cells was predicted to be deactivated (Z-score = −1.043). However, predictions for some canonical pathways remained in the same direction, including neuroinflammation signaling pathway (Z-score = 1.177), Toll-like receptor signaling (Z-score = 2.121), systematic lupus erythematosus in B cell signaling pathway (Z-score = 0.426), and B cell receptor signaling (Z-score = 0.707) (Figure 6D bottom).

### Immune Signatures of Sm-p80-Based Protein Vaccine With GLA-AF

**Figure 7:**
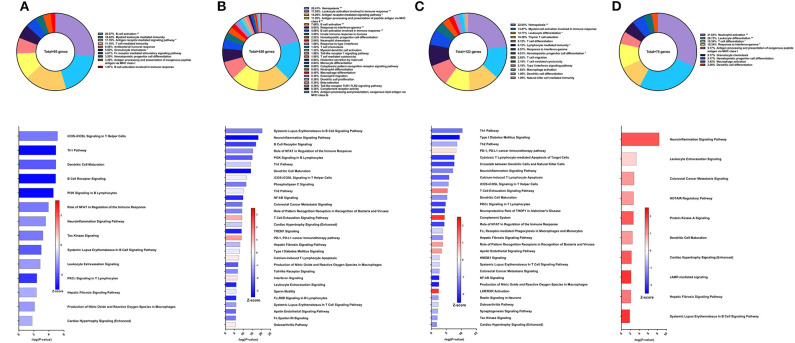
RNA-Seq analysis of rSm-p80+GLA-AF, a recombinant protein vaccine strategy. **(A)** PBMCs after vaccination (Top) Gene ontology enrichment analysis represented as a pie graph of percentages of genes per group out of a total number of differentially expressed genes. “**” and “*” indicates *P* < 0.05 and *P* < 0.01, respectively. (Bottom) Canonical pathway analysis generated using IPA. Bars are plotted based on the – log10(*P*-value) and colored based on predicted activation (red) and deactivation/inhibition (blue) according to the Z-score, a composite assessment based on the degree of overlap between directional expression of genes from the observed data and the Qiagen-curated public database. The top 30 pathways are shown based on the lowest *P*-values. **(B)** PBMCs after challenge (Top) Gene ontology enrichment analysis. (Bottom) Canonical pathway analysis generated using IPA. **(C)** Spleen cells (Top) Gene ontology enrichment analysis. (Bottom) Canonical pathway analysis generated using IPA. **(D)** Mesenteric lymph node cells (Top) Gene ontology enrichment analysis. (Bottom) Canonical pathway analysis generated using IPA.

The sixth vaccine strategy assessed in this study was recombinant Sm-p80 formulated with glucopyranosyl lipid adjuvant (GLA) as an aqueous formulation (AF), resulting in a worm burden reduction of 28.39% (unpublished data). To explore why this strategy had relatively low efficacy, we performed RNA-Seq on PBMCs after vaccination. From the differential expression of 95 genes related to immune system processes, the top GO groups at this time point included B cell activation (26.97%), myeloid leukocyte mediated immunity (18.42%), antigen receptor-mediated signaling pathway (17.76%), and T cell mediated immunity (11.18%) (Figure 7A top). At first glance, these GO groups do not seem vastly different than previous strategies. However, IPA predictions show that the top canonical pathways were all pointing toward deactivation, including iCOS-iCOSL signaling in T helper cells (Z-score = −1.342), Th1 pathway (Z-score = −2.449), dendritic cell maturation (Z-score = −1.633), and B cell receptor signaling (Z-score = −2.449) (Figure 7A bottom).

After parasite challenge, the transcriptome PBMCs reflect a similar immune response to the previous time point, albeit with more DEGs related to immune system processes. These 426 genes are represented largely by the following GO groups: hemopoiesis (20.41%), leukocyte activation involved in immune response (17.05%), antigen receptor-mediated signaling pathway, antigen processing and presentation of peptide antigen via MHC class II (11.28%), and B cell activation (7.68%) (Figure 7B top). While the majority of the top canonical pathways were predicted to be deactivated, some pathways predicted to be activated include T cell exhaustion signaling pathway (Z-score = 1.155), PD-1, PD-L1 cancer immunotherapy pathway (Z-score = 1.387), calcium-induced T lymphocyte apoptosis (Z-score = 0.302), and interferon signaling (Z-score = 0.378) (Figure 7B bottom).

GO enrichment analysis of the spleen cells revealed that the 122 DEGs related to immune system processes belong to GO groups such as hemopoiesis (22.63%), myeloid cell activation involved in immune response (13.87%), leukocyte differentiation (12.77%), thymic T cell selection (10.58%), and response to IFNγ (6.57%) (Figure 7C top). Again, most of the canonical pathways were predicted to be deactivated, including Th1 pathway (Z-score = −1.414), Th2 pathway (Z-score = −0.816), cytotoxic T lymphocyte-mediated apoptosis of target cells (Z-score = −2), and crosstalk between dendritic cells and natural killer cells (Z-score = −1.89). Conspicuously, the complement system was predicted to be activated (Z-score = 2) (Figure 7C bottom).

The mesenteric lymph nodes were processed for RNA-Seq analysis and 75 DEGs were identified that were related to immune system processes. These genes belonged to GO groups such as neutrophil activation (31.03%), leukocyte differentiation (26.72%), T cell differentiation (10.34%), and response to IFNγ (10.34%) (Figure 7D top). Interestingly, pathway analysis with IPA predicted that all canonical pathways related to these DEGs of immune function were activated including neuroinflammation signaling pathway (Z-score = 1.667), leukocyte extravasation signaling (Z-score = 0.447), dendritic cell maturation (Z-score = 1), and hepatic fibrosis signaling pathway (Z-score = 1.342) (Figure 7D bottom).

### Immune Signatures of Sm-p80-Based Protein Vaccine With GLA-Alum

**Figure 8:**
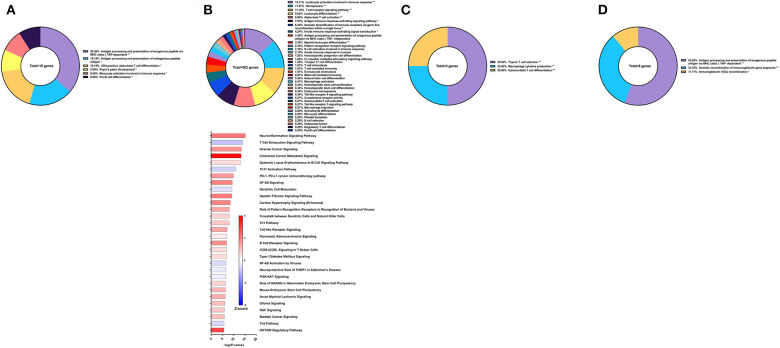
RNA-Seq analysis of rSm-p80+GLA-Alum, a recombinant protein vaccine strategy. **(A)** PBMCs after vaccination (Top) Gene ontology enrichment analysis represented as a pie graph of percentages of genes per group out of a total number of differentially expressed genes. “**” and “*” indicates *P* < 0.05 and *P* < 0.01, respectively. (Bottom) Canonical pathway analysis generated using IPA. Bars are plotted based on the – log10(*P*-value) and colored based on predicted activation (red) and deactivation/inhibition (blue) according to the Z-score, a composite assessment based on the degree of overlap between directional expression of genes from the observed data and the Qiagen-curated public database. The top 30 pathways are shown based on the lowest *P*-values. **(B)** PBMCs after challenge (Top) Gene ontology enrichment analysis. (Bottom) Canonical pathway analysis generated using IPA. **(C)** Spleen cells (Top) Gene ontology enrichment analysis. (Bottom) Canonical pathway analysis generated using IPA. **(D)** Mesenteric lymph node cells (Top) Gene ontology enrichment analysis. (Bottom) Canonical pathway analysis generated using IPA.

Historically, we have observed that a mixed and balanced immune response between Th1, Th2, and Th17 provided the highest levels of protection, aligning with studies done by others (37, 38). Hence, with aluminum hydroxide (Alum) as the adjuvant, we sought to explore whether tuning the immune response toward a balanced response would enhance protection against *S. mansoni*. We previously reported that this recombinant protein vaccine strategy provided 38.53% reduction in worm burden in baboons compared to the control group which only received the adjuvant alone (32). Surprisingly, only 10 genes related to immune system processes were differentially expressed in the PBMCs after vaccination. These genes fell under the following GO groups: antigen processing and presentation of exogenous peptide antigen via MHC class I, TAP-dependent (36.36%), CD4-positive, alpha-beta T cell differentiation (18.18%), antigen processing and presentation of endogenous peptide antigen (18.18%), and others. Pathway analysis with IPA did not reveal any significant predictions for canonical pathways related to the immune response at this time point (Figure 8A).

Following parasite challenge and disease progression, 502 DEGs related to immune system processes were identified in the PBMCs, a drastic increase from the previous time point. The majority of these DEGs were categorized in GO groups such as leukocyte activation involved in immune response (13.11%), hemopoiesis (11.97%), T cell receptor signaling pathway (11.10%), leukocyte differentiation (9.62%), alpha-beta T cell activation (8.94%), antigen immune response-activating signaling pathway (7.67%), and many others (Figure 8B top). Pathway analysis using IPA revealed nuanced predictions for the immune response, including activation of the Th1 pathway (Z-score = 0.832) and deactivation for the Th2 pathway (Z-score = −0.632) and Th17 pathway (Z-score = −0.728) (Figure 8B bottom).

The spleen and mesenteric lymph nodes did not reveal many differentially expressed immune genes with a total of only 3 and 8 identified, respectively. For the spleen cells, these 3 DEGs related to immune system processes were placed into 3 groups: thymic T cell selection (50.0%), macrophage cytokine production (25.0%), and gamma-delta T cell differentiation (25.0%) (Figure 8C). The eight DEGs from the lymph node cells were also represented with 3 groups: antigen processing and presentation of exogenous peptide antigen via MHC class I, TAP-dependent (55.56%), somatic recombination of immunoglobulin gene segments (33.33%), and immunoglobulin V(D)J recombination (11.11%) (Figure 8D). IPA was unable to predict the direction of any pathways for the DEGs from the spleen and lymph nodes for this vaccine strategy.

### Immune Signatures of Sm-p80-Based Protein Vaccine With GLA-SE

**Figure 9:**
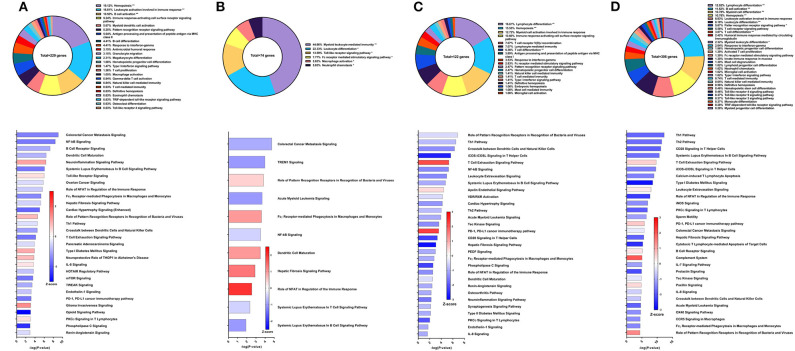
RNA-Seq analysis of rSm-p80+GLA-SE, a recombinant protein vaccine strategy. **(A)** PBMCs after vaccination (Top) Gene ontology enrichment analysis represented as a pie graph of percentages of genes per group out of a total number of differentially expressed genes. “**” and “*” indicates *P* < 0.05 and *P* < 0.01, respectively. (Bottom) Canonical pathway analysis generated using IPA. Bars are plotted based on the – log10(*P*-value) and colored based on predicted activation (red) and deactivation/inhibition (blue) according to the Z-score, a composite assessment based on the degree of overlap between directional expression of genes from the observed data and the Qiagen-curated public database. The top 30 pathways are shown based on the lowest *P*-values. **(B)** PBMCs after challenge (Top) Gene ontology enrichment analysis. (Bottom) Canonical pathway analysis generated using IPA. **(C)** Spleen cells (Top) Gene ontology enrichment analysis. (Bottom) Canonical pathway analysis generated using IPA. **(D)** Mesenteric lymph node cells (Top) Gene ontology enrichment analysis. (Bottom) Canonical pathway analysis generated using IPA.

Recombinant Sm-p80 formulated with GLA-SE (31) demonstrated the highest levels of protection among the vaccine strategies tested. The latest studies using this vaccine strategy was reported to have female worm reduction of 93.35% and overall worm burden reduction of 65.90%. To examine the effects of the vaccine, we performed RNA-Seq analysis on PBMCs after the vaccination regimen. We identified 229 DEGs related to immune system processes. These genes were found in GO groups such as hemopoiesis (19.12%), leukocyte activation involved in immune response (16.81%), B cell activation (10.5%), and others (Figure 9A top). Eight of the top 30 canonical pathways in terms of statistical significance were predicted to be activated, including neuroinflammation signaling pathway (Z-score = 1.069), Toll-like receptor signaling (Z-score = 0.378), and IL-8 signaling (Z-score = 0.333). Among the canonical pathways predicted to be deactivated were colorectal cancer metastasis signaling (Z-score = −1.5), NF-kB signaling (Z-score = −1.604), B cell receptor signaling (Z-score = −1), dendritic cell maturation (Z-score = −0.905), and the Th1 pathway (Z-score = −0.378) (Figure 9A bottom).

Prior to sacrifice, fewer genes were differentially expressed in the PBMCs. These 74 DEGs related to immune system processes were categorized in GO groups such as myeloid leukocyte mediated immunity (44.66%), leukocyte differentiation (22.33%), Toll-like receptor signaling pathway (13.59%), and Fc receptor-mediated stimulatory signaling pathway (7.77%) (Figure 9B top). Accordingly, fewer pathways were able to be predicted with statistical significance. Canonical pathways that were predicted to be activated include role of PRR in recognition of bacteria and viruses (Z-score = 0.378), FCγ receptor-mediated phagocytosis in macrophages and monocytes (Z-score = 0.447), dendritic cell maturation (Z-score = 1), hepatic fibrosis signaling pathway (Z-score = 1.342), and role of NFAT in regulation of the immune response (Z-score = 2) (Figure 9B bottom).

Spleen cells were processed for RNA-Seq and 122 DEGs related to immune system processes were accepted after cutoffs. These DEGs were represented in the following GO groups: lymphocyte differentiation (16.61%), hemopoiesis (15.55%), myeloid cell activation involved in immune response (12.72%), T cell differentiation (6.36%), and others (Figure 9C top). Only 3 of the top 30 canonical pathways were predicted to be activated according to pathway analysis by IPA: T cell exhaustion, apelin endothelial signaling pathway, and PD-1, PDL1 cancer immunotherapy pathway. Some notable pathways that were predicted to be deactivated include the Th1 pathway (Z-score = −0.816), Th2 pathway (Z-score = −0.816), and pathways related to PKCθ signaling such as NF-kB signaling (Z-score = −1.414), CD28 signaling (Z-score = −2.236), iCOS-iCOSL signaling (Z-score = −2.646), and role of NFAT (Z-score = −1.342) (Figure 9C bottom).

The mesenteric lymph node cells yielded 306 DEGs related to immune system processes. The largest GO groups for these DEGs include lymphocyte differentiation (12.92%), B cell activation (11.52%), myeloid cell differentiation (10.78%), and hemopoiesis (10.78%) (Figure 9D top). Many canonical pathways were predicted to have the same direction as those for spleen cells, including Th1 pathway (Z-score = −1.807), Th2 pathway (Z-score = −1.732), and pathways related to PKCθ signaling. Notably, the complement system was predicted to be activated in the lymph node cells with this strategy (Z-score = 2) (Figure 9D bottom).

## Discussion

The complexity of schistosomiasis, socio-economic implications notwithstanding, demand nuance, and discretion to solve. Schistosomes have co-existed and co-evolved with mankind for millennia and they invariably have honed their abilities to evade attacks by our immune response. Indeed, schistosomes do not simply avoid detection but can thrive in their host for decades (39), actively modulating our immune response to propagate their life cycle (40). Two of the “grails” of vaccinology for schistosomiasis would be to find a universal immune signature to predict protection or to define a surrogate of protection. As of yet, no universal immune signature nor surrogate of protection has been defined or agreed upon for schistosomiasis.

As a follow-up from eight previous vaccine studies, we assessed the killing effect of immune sera from Sm-p80-vaccinated baboons on *S. mansoni* schistosomula *in vitro*. Our results showed a significant schistosomula killing when cultured in the presence of sera obtained from Sm-p80-vaccinated baboons when compared to sera from control baboons and the killing effect was significantly augmented with the addition of exogenous complement. Our data also showed that the complement effect was completely reversed when heat inactivated. Cumulatively, these data strongly suggest possible roles for Sm-p80-specific antibodies, complement and/or both in vaccine-mediated parasite killing. The parasite killing observed is not surprising as we have previously shown that Sm-p80 protein is highly expressed on the surface of *S. mansoni* schistosomula (25), presenting multiple target epitopes for antibodies. We further explored the *in vivo* protective role of these Sm-p80 antibodies against *S. mansoni* in a mouse model of infection and disease. In the experimental group, heterologous transfer of purified IgG from baboons immunized with Sm-p80-based vaccine conferred significant protection in mice following *S. mansoni* cercarial challenge. Specifically, we observed a 59% reduction in total worm numbers from experimental mice that received purified Sm-p80-specific IgG when compared to the control mice. More importantly, there was a 60% reduction in total egg-producing female worms from these experimental mice suggesting that Sm-p80-specific IgG might be selectively killing female worms. This is in agreement with our previous preclinical efficacy study in which we also observed significant female worm killing in baboons immunized with Sm-p80 vaccine (32).

The pathology of schistosomiasis and its severity is directly linked to the number of schistosome eggs trapped within the host tissues, particularly the liver, and the host immune responses to antigens secreted by viable eggs within these tissues (3, 41). In this study, we found that passively transferred Sm-p80-specific IgG lead to a significant reduction in hepatic and intestinal egg load in mice compared the control group, demonstrating anti-pathology effect of anti-Sm-p80-specific antibodies. In addition to the observed reduction in tissue egg burden, we also observed a significant reduction in egg viability/hatching rates in eggs obtained from the liver and a moderate reduction in egg hatching rates in eggs obtained from the intestines. Specifically, liver and intestinal eggs recovered from experimental mice had a 49.6 and 42.8% reduction in hatching rates, respectively. Taken together, the data presented here on Sm-p80-specific IgG-mediated female worm killing, reduction in tissue egg burden, and reduction in egg hatching highlight not only the anti-pathology effect of Sm-p80 antibodies but also its potential at reducing transmission as fewer viable eggs are released into the environment (42, 43).

Exploration of the immune response through the lens of transcriptomic analyses revealed a kaleidoscopic range of results. Initially, we overlaid the DEGs from each vaccine strategy to determine whether we could identify any genes that would be differentially expressed consistently. While we certainly found common DEGs between various strategies, no DEGs were common among all eight strategies (Supplementary Figure 33). We subsequently examined the relationship between the DEGs of various strategies with their respective GO groups. Categories of gene ontology displayed some similarity. For example, we found evidence for prominent T cell and B cell responses in every strategy; while particular components of the immune response may not be reflected on GO groups in one tissue, they would appear in another. Interestingly, we found that hemopoiesis was one of the largest GO groups for each time point and tissue type, appearing in seven out of the eight strategies in the PBMCs after vaccination. After parasite challenge, we found that the hemopoiesis GO group was more represented in the spleen and lymph nodes compared to PBMCs. Hemopoiesis, defined broadly as the process by which immune progenitor cells develop into mature cells with varying though distinct lineages, has implications for vaccine-based immune responses and subsequent protective efficacy to said vaccines (44, 45). Perturbations in the immune response *via* vaccines or pathogens elicit cytokines, growth factors, and other signaling pathways that affect the regulation of hematopoietic stems cells (HSCs) (46). Based on differential expression of members of the Wnt family, the Wnt signaling cascade may be a key regulator for HSCs and hemopoiesis in response to Sm-p80-based vaccines, although the exact mechanism linking hemopoiesis to schistosome worm burden reduction is unknown.

We observed much evidence for involvement of cellular immune responses, especially DEGs related to T cells, corroborating our previous reporting based on analyses with PCR, ELISPOT, and flow cytometry (22, 30–32). Through RNA-Seq analyses, we have found that protein Kinase C-theta (PKCθ) signaling in T helper cells play a role in Sm-p80-induced cellular immunes. PKCθ has been described to balance regulatory T cell (T_regs_) and effector T cell functions through a range of signaling cascades (47, 48). These signaling cascades include CD28 signaling (49), iCOS-iCOSL signaling (50), NFAT (51), and NF-kB (52). CD28 is a costimulatory factor critical for the induction of MHC class II-restricted T cell responses and bridges the humoral response through CD80 (53, 54). Indeed, others have shown that CD28 costimulation is important in mounting an appropriate response to *S. mansoni* infection through the use of CD28-deficient (–/–) mice which produced reduced levels of parasite-specific IgG1 and IgE antibodies compared to wild-type animals (55). The inducible co-stimulator (iCOS) and its ligand, iCOSL, have a range of functions that include the regulation of T helper cells, cytokine production, T cell/ B cell collaboration through CD40/CD40L pathway, and immunoglobulin class switching (56, 57). While the iCOS-iCOSL pathway has been reported to have an effect on fibrosis and hepatopathology in mice infected with schistosomiasis, its exact role is debated (58, 59). The nuclear factor of activated T cells (NFAT) pathway is known to regulate T cell activation, differentiation, and development (60). Others have demonstrated that NFAT^−/−^ mice have increased eosinophil and serum levels of IgE (61) and that NFAT family proteins positively regulate IL-4 production (62), suggesting that NFAT may play a role in the Th2 response in schistosomiasis (63, 64). NF-kB are among transcription factors that induce IL-2 expression and regulates inflammatory responses (65). Others have shown that *S. mansoni* interferes with the NF-kB pathway, thereby disrupting the recruitment of leukocyte recruitment to the lungs and allowing the parasite to evade the immune response (66). Additionally, it has been suggested that manipulation of NF-kB signaling may be a method of treating schistosomiasis (67–69). Although evidence for these pathways were frequently observed from the RNA-Seq analysis of the eight different Sm-p80-based vaccine strategies, their predictions were variable and inconsistent between strategies.

Ultimately, what we discovered may be unsurprising – Sm-p80 vaccine-induced signatures of immunity differ based on the strategy and/or adjuvant used and no universal signatures of immunity were found. While common pathways were found in each vaccine strategy, the direction and magnitude of predictions for these pathways differed without obvious patterns. This conclusion may be due to several factors to consider. First, the biological diversity in our studies presents a dichotomy of benefits and detriments. The baboons used in Sm-p80-based vaccine studies were outbred with ages that ranged from 1 year old to 15 years old. Introduction of biological diversity in our studies would better recapitulate results when we transition into human clinical trials where the extent of diversity would increase. However, utilizing outbred animal models introduce statistical variance which reduces our ability to distinguish more subtle immune signatures in which high fold change is biologically unreasonable to expect. Furthermore the timing of when samples were collected was optimized for assessment of antibody responses. Vaccine-induced changes in gene transcription detectable by RNA-Seq occur as early as one day after immunization (70–72) yet ethical considerations prevent us from frequent blood draws or early sacrifices for sample collection. Nevertheless, systems biology approaches have proven to be valuable tools in our understanding of the global transcriptomic effects of Sm-p80-induced protection. Combined with the previously published results from conventional immunological techniques, Sm-p80-based vaccines have consistently demonstrated robust immune responses associated with protection and is poised to enter Phase I human clinical trials.

## Data Availability Statement

The datasets generated for this study can be found in the NCBI database with the BioProject ID PRJNA623028.

## Ethics Statement

The animal study was reviewed and approved by TTUHSC IACUC Protocol Number 20010202.

## Author Contributions

AM, WZ, JF, SKh, SS, JS, and AJS completed the *in vitro* and *in vivo* mouse studies. WZ, LL, GA, WT, SKa, JR, DCare, RW, JP, and RD contributed to the baboon studies. WZ, AJS, and SL completed the RNA extraction for library preparation and RNA-seq. WZ, LL, and AR completed the RNA-seq data analysis. AM and JS performed gene validation. LL, AM, and WZ performed other data analysis. LL and AM wrote the manuscript. SG, FM, and DCart provided edits, revisions, and study support. AAS conceived and designed the study. All authors contributed to the article and approved the submitted version.

## Conflict of Interest

DCart and SG were employed by PAI Life Sciences, Seattle, WA, USA. The remaining authors declare that the research was conducted in the absence of any commercial or financial relationships that could be construed as a potential conflict of interest.
