# Supplementary material for: Fifteen Years of Sm-p80-Based Vaccine Trials in Nonhuman Primates: Antibodies From Vaccinated Baboons Confer Protection in vivo and in vitro From Schistosoma mansoni and Identification of Putative Correlative Markers of Protection
Source: Front Immunol. 2020 Jun 19;11:1246. doi: 10.3389/fimmu.2020.01246 (PMC7318103; doi:10.3389/fimmu.2020.01246)
Supplement: Supplementary file 1 [file Data_Sheet_1.docx]

**Fifteen years of Sm-p80-based vaccine trials in nonhuman primates: Antibodies from vaccinated baboons confer protection *in vivo* and *in vitro* from *Schistosoma mansoni* and identification of predictive correlates of protection markers**

**Supplementary Materials**

**Supplementary Table 1. Primer sequences for select genes.**

| Gene name | Primer Sequences 5' - 3' |
| --- | --- |
| bAXL | AAGTGTTCGAGCCAACAGTG |
|  | TGTTCAAGGTGGCTTCAGTG |
| bPRAM1 | TCAAAGCGCTCTCCAAGAAG |
|  | TTCAGGCTGTGAGAACTTGC |
| bBST1 | TTTCTTGAGCTGGTGTCGAC |
|  | TACTGGATGGATGCCCTTTTCC |
| bPF4 | GTGTGTGAAGACCACCTCCC |
|  | TTTTCTTGTACAGCGGGGCT |
| bTLR7 | GATCGTGGACTGCACAGACA |
|  | AGTCTGTGAAAGGACGCTGG |
| bLAT | AGCCGGGAGTATGTGAATGTG |
|  | AGTTCAGCTCCTGCAGATTCTC |
| bTST | TGGATGTTCCGTGTGTTTGG |
|  | TTTGAAAACGGCCGGTTCTG |
| bLMO1 | TAACGTGTATCACCTCGACTGC |
|  | TCAAAGGTGCCATTGAGCTG |
| bDAB2 | ACAACCGGGCATTTGGTTAC |
|  | TGTTGCCCGGTTTTTATGGC |
| hIGKV3-16 | GCAGCCTCTGGATTCACCTT |
|  | CGGCTCTCCGTCTGTTCTTT |
| bPLTP | TTCTCTCCACGTTCATCACCTC |
|  | ATGCCAACAAGCTCATCCAC |
| hIGV1-24 | TGCAAGGTTTCCGGATACAC |
|  | AGGATCAAAACCTCCCATCCAC |
| hIGLV4-3 | AGGAGCTTCAAGCCAACAAG |
|  | TGCTTTGTTTGGAGGGTGTG |
| bCXCR3 | GACCACCAAGTGCTAAATGACG |
|  | TCGAAGTTCAGGCTGAAGTCC |
| bGATA3 | CCAGACCAGAAACCGGAAAATG |
|  | CGGGTTAAACGAGCTGTTCTTG |
| hIGKV1-12 | AAAGCCCCTAAGCTCCTGATC |
|  | ACTGCCGCTGAACCTTGATG |
| bCD3G | TTGCTGGACAGGATGGAGTTC |
|  | TAGAGCTGGTCATTGGGCAAC |

**Supplementary Table 2. qPCR validation of select genes.**

| Gene name | Strategy | Tissue | qPCR Log2(FC) | RNA-Seq Log2(FC) |
| --- | --- | --- | --- | --- |
| bAXL | Sm-p80-VR1020 | PBMCs - after vaccination | -0.5785338 | -0.590149209 |
|  | Sm-p80-VR1020 + rSm-p80+ODN10104 | PBMCs - after vaccination | 0.43379847 | 1.214904374 |
|  | Sm-p80-VR1020 + rSm-p80+Resiquimod | PBMCs - after vaccination | -1.1811721 | -0.602764742 |
| bPRAM1 | Sm-p80-VR1020 | PBMCs - after vaccination | 0.14027341 | -0.708202915 |
|  | Sm-p80-VR1020 + rSm-p80+ODN10104 | PBMCs - after vaccination | 0.88173993 | 1.30935639 |
|  | Sm-p80-VR1020 + rSm-p80+Resiquimod | PBMCs - after vaccination | -0.313441 | 1.149887573 |
| bBST1 | Sm-p80-VR1020 | PBMCs - after challenge | 1.00909678 | -0.805829241 |
|  | Sm-p80-VR1020 + rSm-p80+ODN10104 | PBMCs - after challenge | 0.24715551 | 0.916557002 |
|  | Sm-p80-VR1020 + rSm-p80+Resiquimod | PBMCs - after challenge | -0.3607833 | 0.950965818 |
| bPF4 | rSm-p80+GLA-SE | PBMCs - after vaccination | 0.89321009 | 1.71433089 |
|  | rSm-p80+GLA-AF | PBMCs - after vaccination | -4.7362633 | 1.222361489 |
| bTLR7 | rSm-p80+GLA-SE | PBMCs - after vaccination | -0.7893213 | -0.643520882 |
|  | rSm-p80+GLA-AF | PBMCs - after vaccination | 0.09919484 | -0.871763611 |
| bLAT | rSm-p80+ODN10104 | PBMCs - after challenge | 0.82688522 | -0.721054416 |
|  | rSm-p80+Resiquimod | PBMCs - after challenge | 1.08713849 | -0.59484946 |
|  | rSm-p80+GLA-SE | PBMCs - after challenge | 0.25970968 | 0.654568983 |
|  | rSm-p80+GLA-AF | PBMCs - after challenge | -0.9668007 | 1.070960842 |
|  | rSm-p80+GLA-Alum | PBMCs - after challenge | -0.5213706 | -0.894121191 |
| bTST | rSm-p80+GLA-SE | Spleen cells | -0.2965449 | 0.746619644 |
|  | rSm-p80+GLA-AF | Spleen cells | -1.1814766 | -0.642806412 |
| bLMO1 | rSm-p80+GLA-SE | Spleen cells | -0.2317521 | -0.718497846 |
|  | rSm-p80+GLA-AF | Spleen cells | 1.16630109 | -0.604618182 |
| bDAB2 | rSm-p80+GLA-SE | Spleen cells | 0.06149673 | 0.680080583 |
|  | rSm-p80+GLA-AF | Spleen cells | 0.45524693 | 0.811551545 |
| hIGKV3-16 | rSm-p80+GLA-SE | Spleen cells | -0.90185 | -0.794977092 |
|  | rSm-p80+GLA-AF | Spleen cells | -1.0619024 | -0.753066853 |
| bPLTP | rSm-p80+GLA-SE | Spleen cells | 0.81885056 | 0.715944381 |
|  | rSm-p80+GLA-AF | Spleen cells | 1.42958919 | 0.636029303 |
| hIGV1-24 | rSm-p80+GLA-SE | Spleen cells | -7.0023102 | -0.935617563 |
|  | rSm-p80+GLA-AF | Spleen cells | 0.51460203 | -0.806646424 |
| hIGLV4-3 | rSm-p80+GLA-SE | Spleen cells | -0.9988463 | 0.835906408 |
|  | rSm-p80+GLA-AF | Spleen cells | 0.26747587 | 0.856073235 |
| bCXCR3 | rSm-p80+GLA-SE | Spleen cells | -2.584674 | -0.594977224 |
|  | rSm-p80+GLA-AF | Spleen cells | 0.93266595 | -0.778090223 |
| bGATA3 | rSm-p80+GLA-SE | Spleen cells | -1.7046296 | -0.689250046 |
|  | rSm-p80+GLA-AF | Spleen cells | 1.98207178 | -0.588864978 |
| hIGKV1-12 | rSm-p80+GLA-SE | Spleen cells | -2.69619 | 0.769541532 |
|  | rSm-p80+GLA-AF | Spleen cells | -0.5292809 | -0.724944558 |
| bCD3G | rSm-p80+GLA-SE | Lymph node cells | 1.24114927 | 0.80261031 |
|  | rSm-p80+GLA-AF | Lymph node cells | 1.10057404 | 2.018288984 |
|  | rSm-p80+GLA-Alum | Lymph node cells | -1.7607242 | 0.926977395 |

**Supplementary Figure 1. GO term network: Sm-p80-VR1020 - after vaccination.**

**
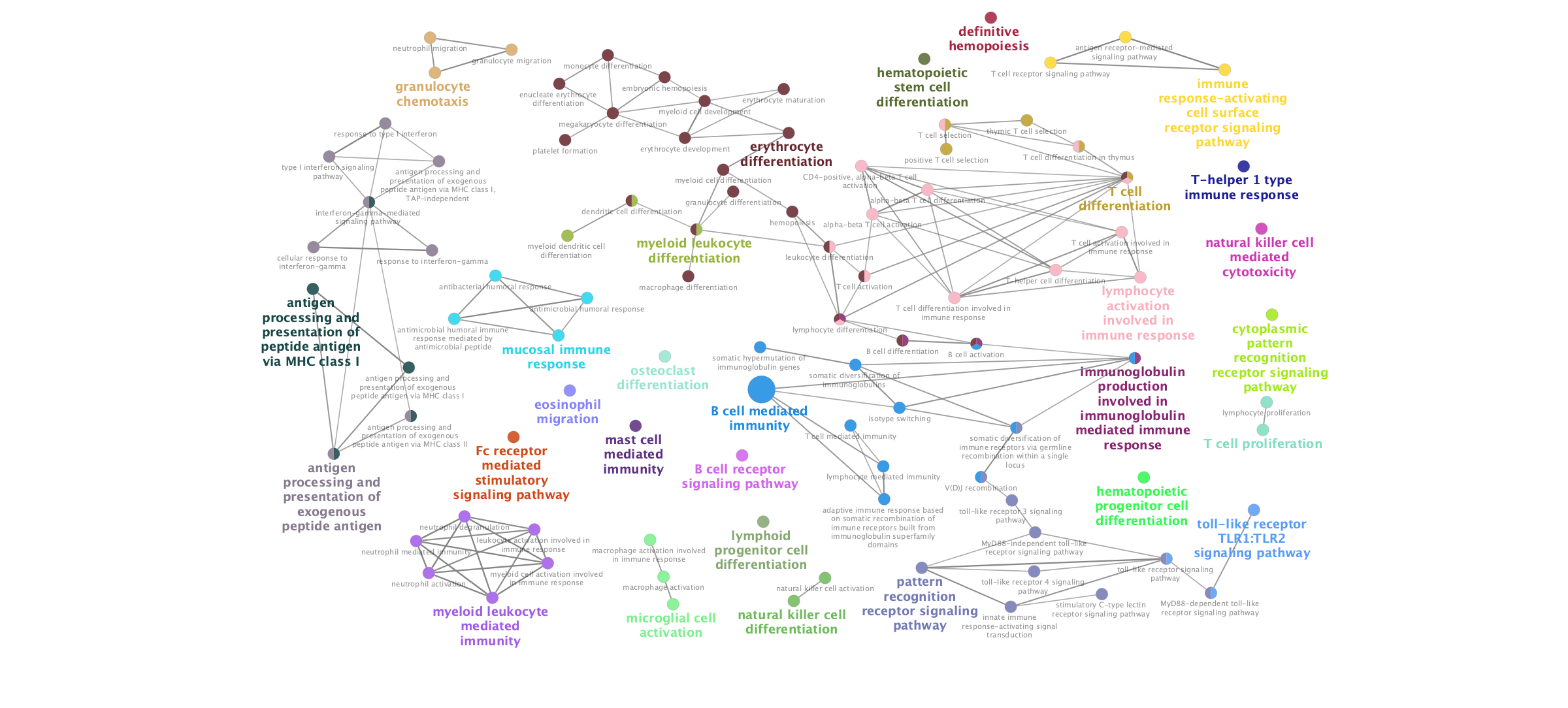
**

**Supplementary Figure 2. GO term network: Sm-p80-VR1020 - after challenge.**

**
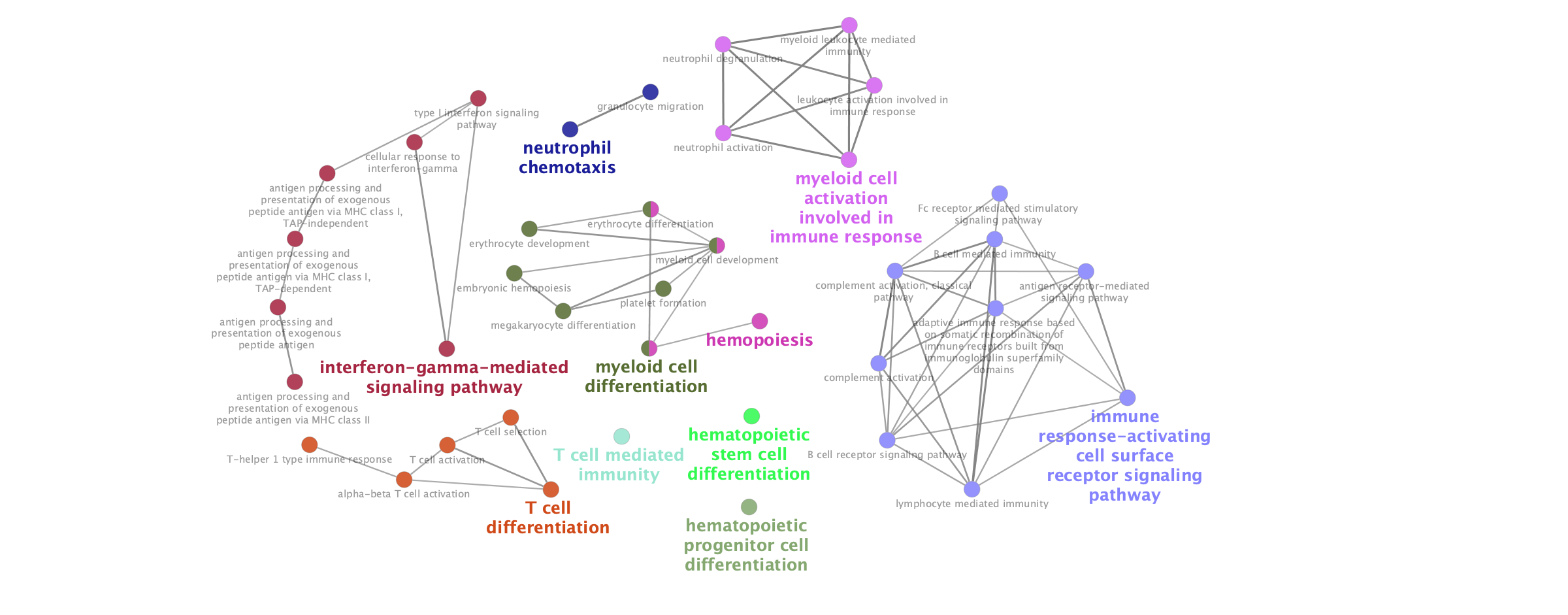
**

**Supplementary Figure 3. GO term network: Sm-p80-VR1020 - spleen cells.**

**
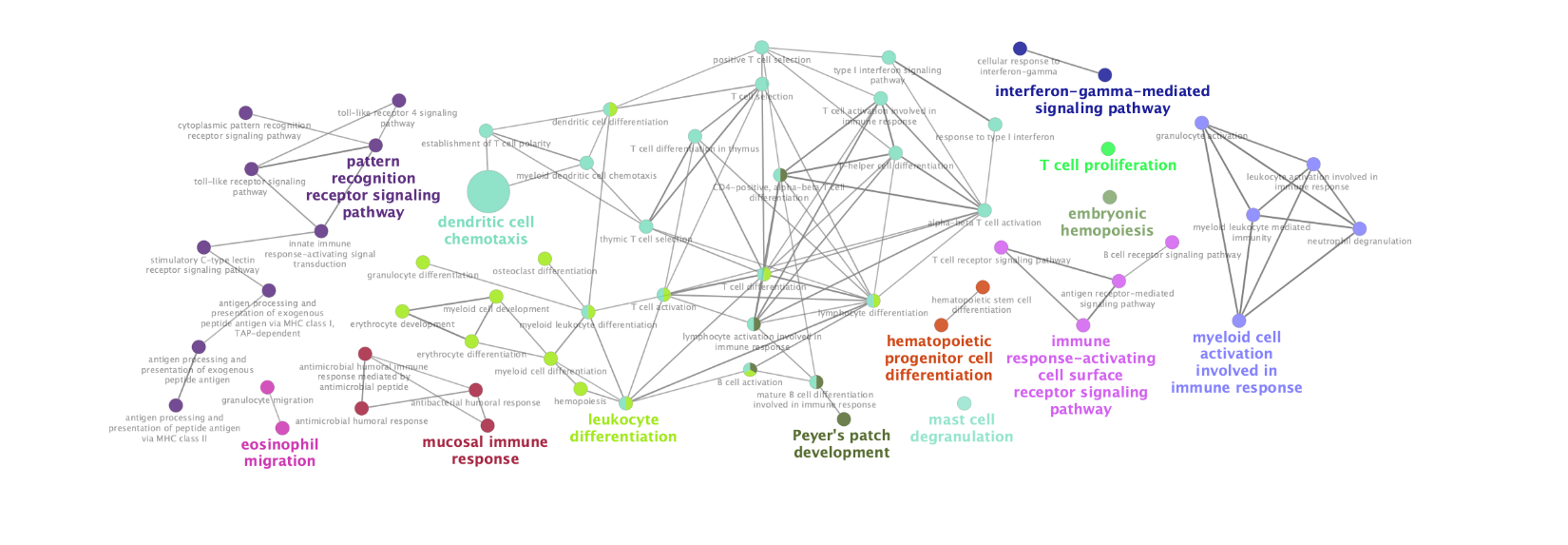
**

**Supplementary Figure 4. GO term network: Sm-p80-VR1020 - lymph node cells.**

**
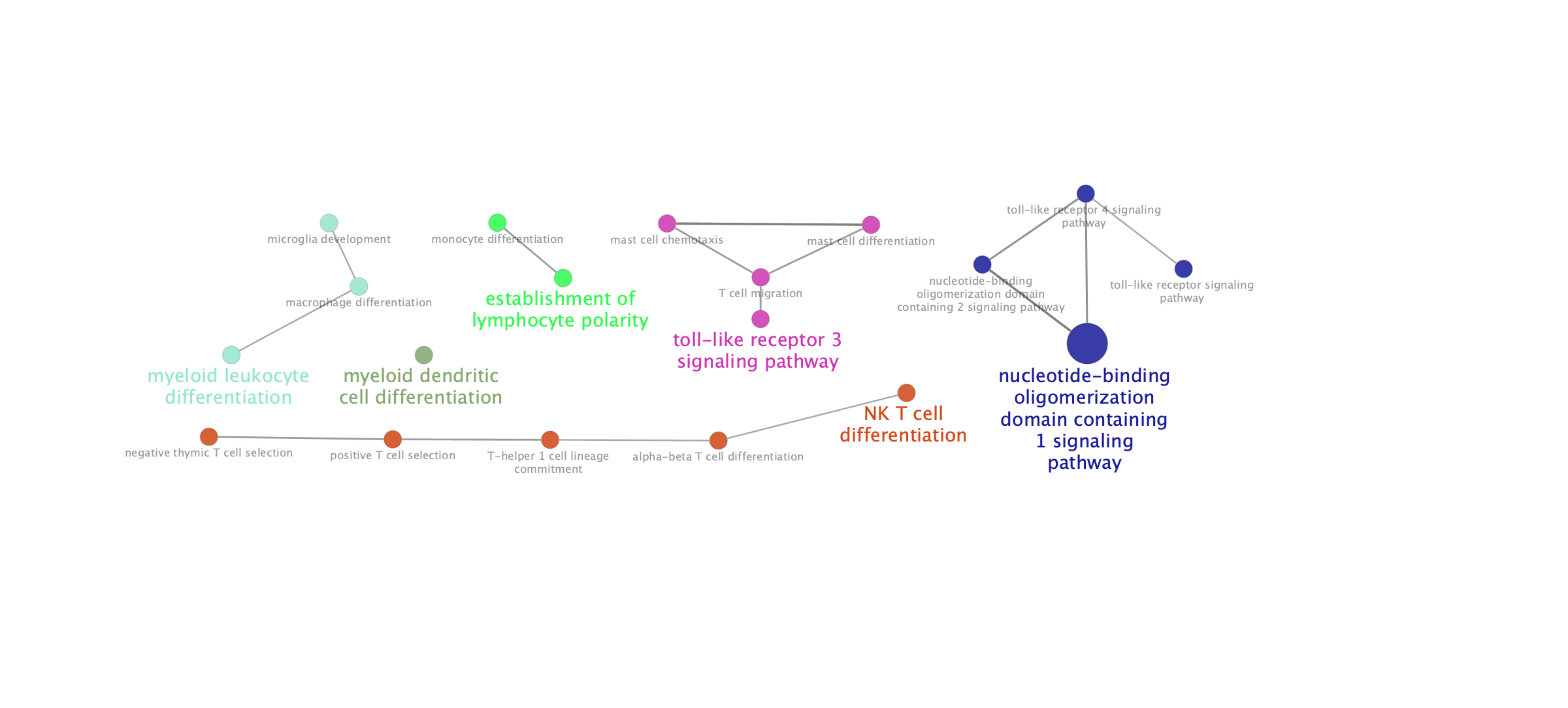
**

**Supplementary Figure 5. GO term network: Sm-p80-VR1020 + rSm-p80+ODN10104 - after vaccination.**

**
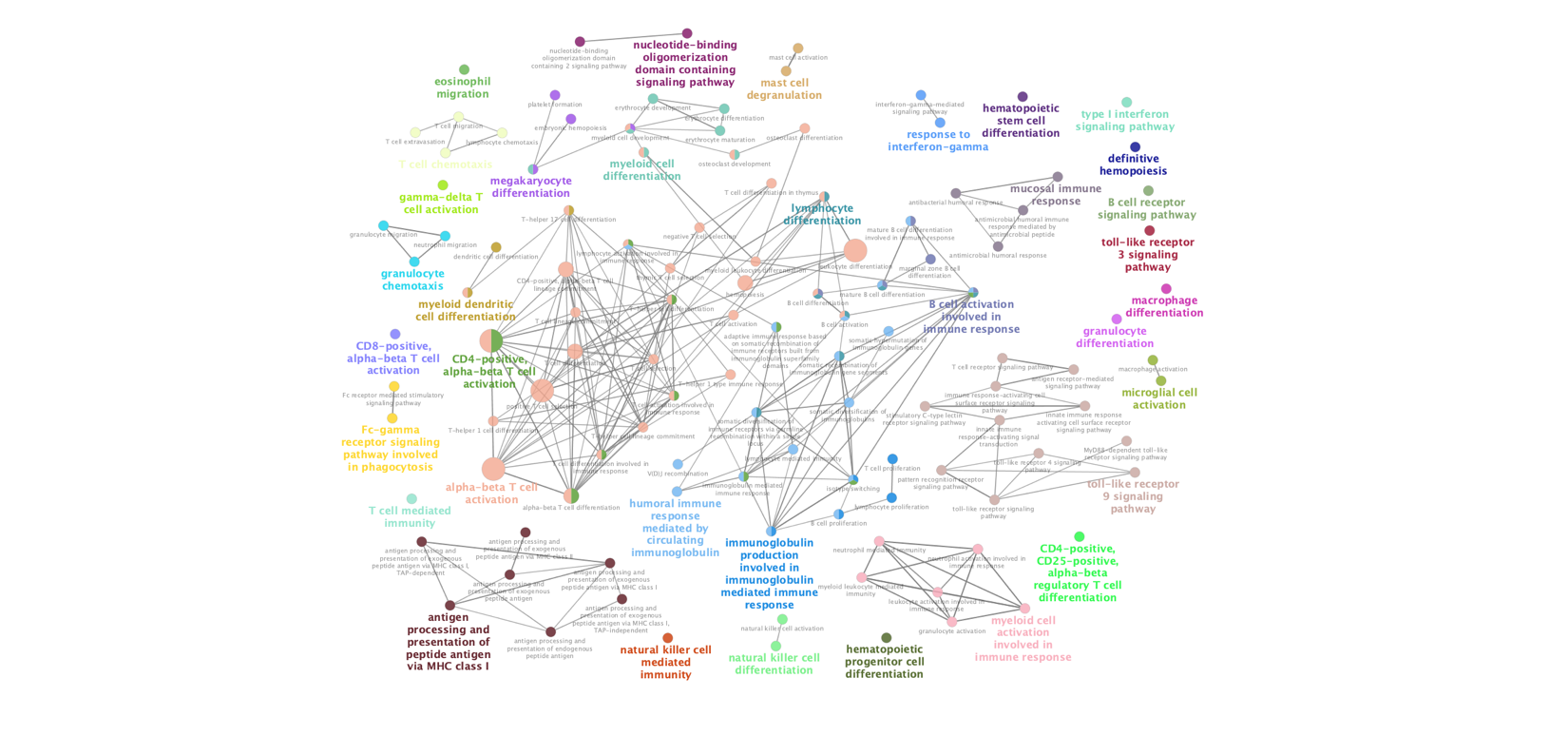
**

**Supplementary Figure 6. GO term network: Sm-p80-VR1020 + rSm-p80+ODN10104 - after challenge.**

**
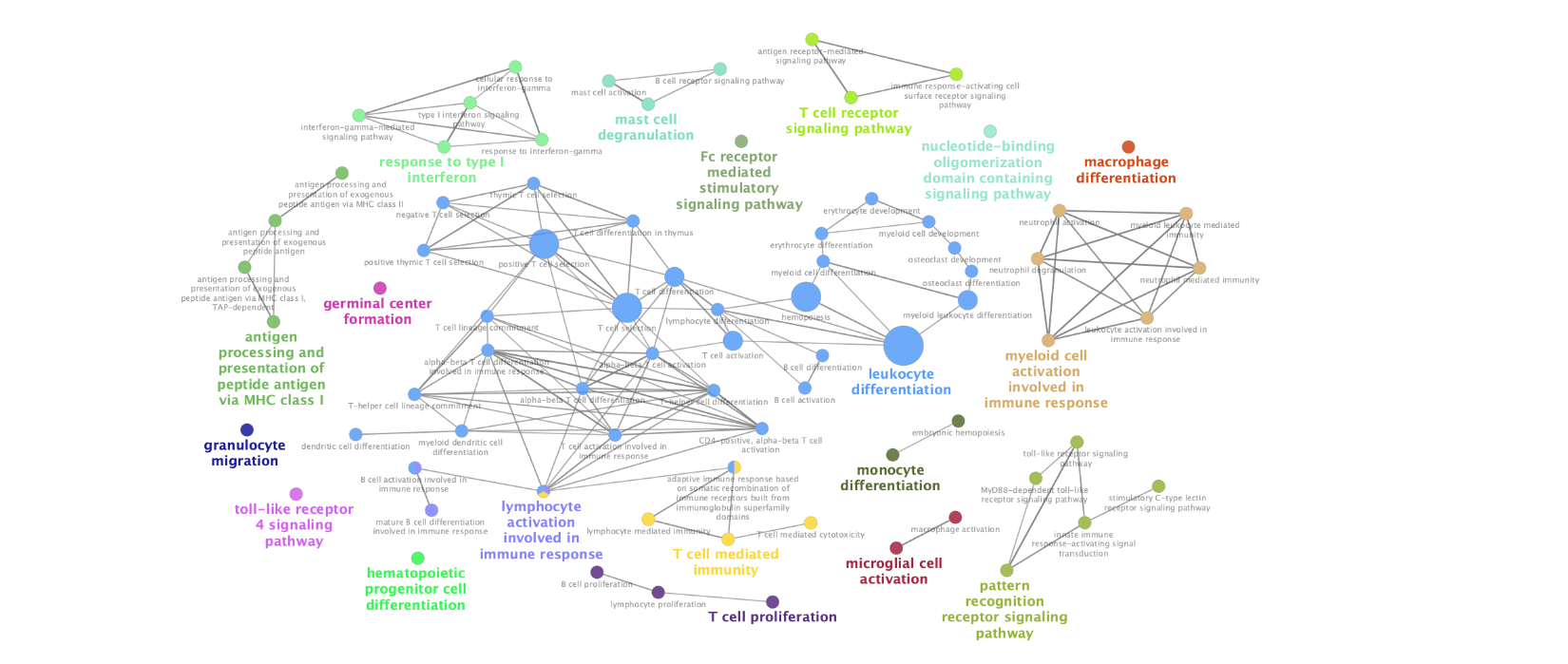
**

**Supplementary Figure 7. GO term network: Sm-p80-VR1020 + rSm-p80+ODN10104 - spleen cells.**

**
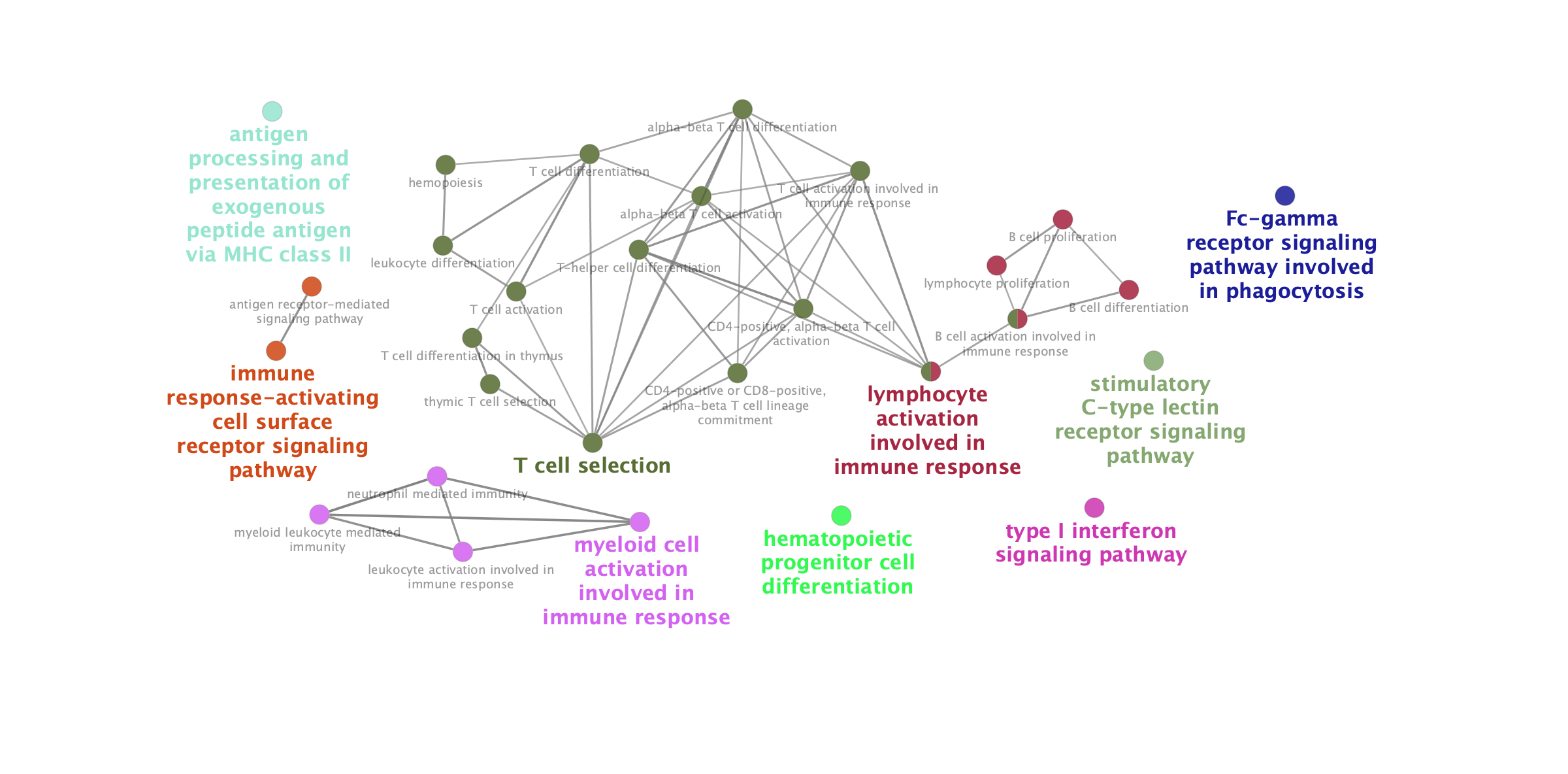
**

**Supplementary Figure 8. GO term network: Sm-p80-VR1020 + rSm-p80+ODN10104 - lymph node cells.**

**
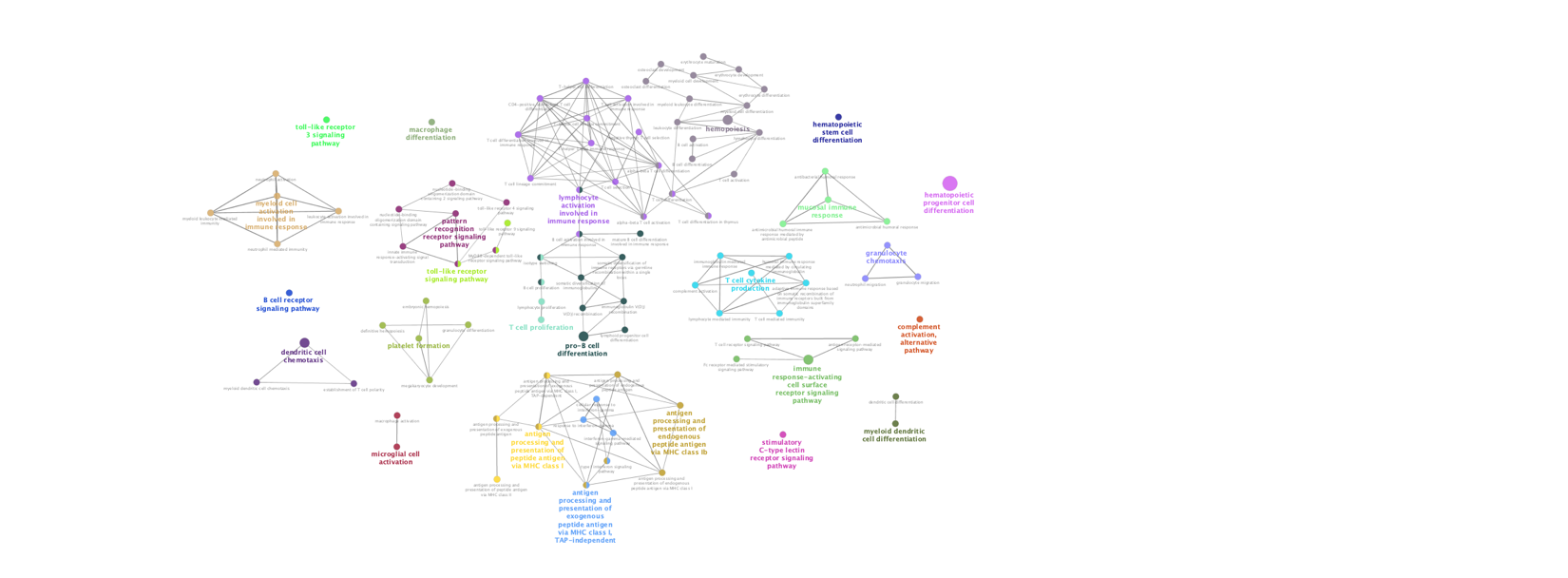
**

**Supplementary Figure 9. GO term network: Sm-p80-VR1020 + rSm-p80+Resiquimod - after vaccination.**

**
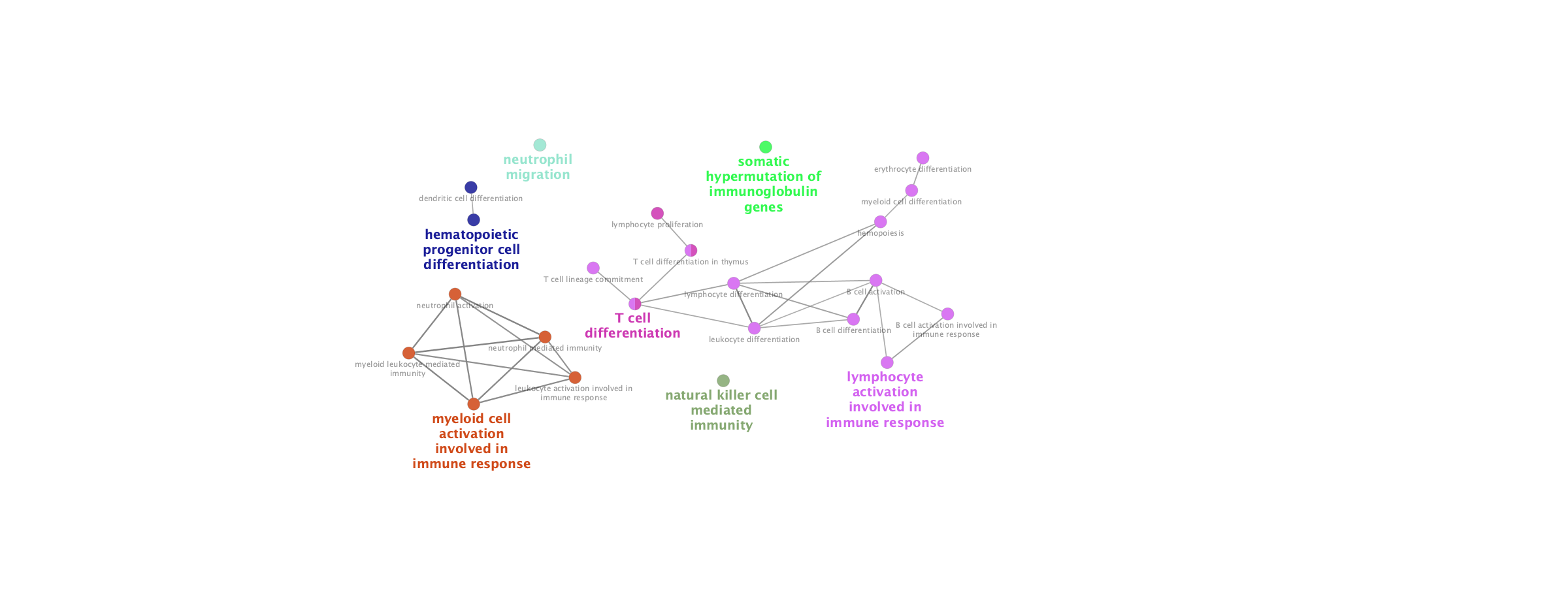
**

**Supplementary Figure 10. GO term network: Sm-p80-VR1020 + rSm-p80+Resiquimod - after challenge.**

**
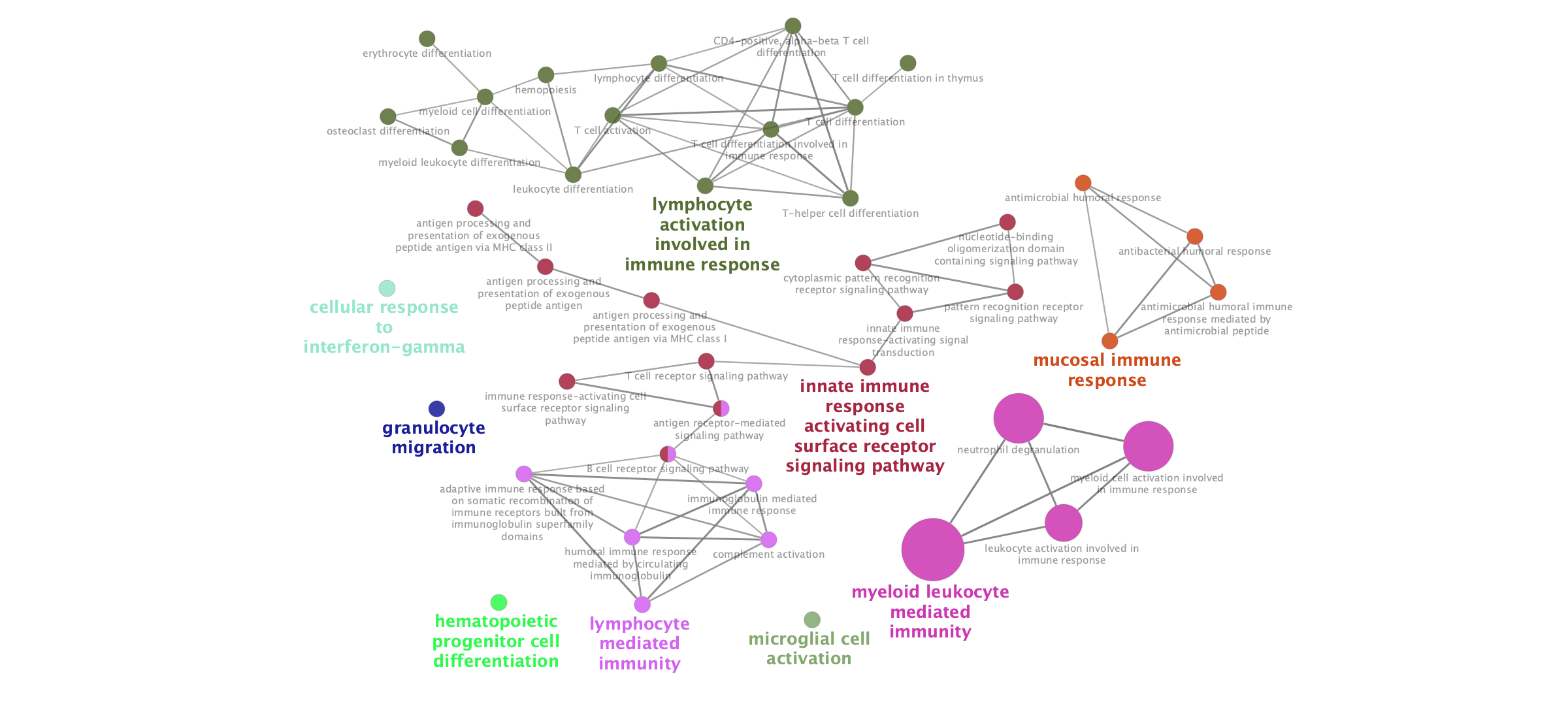
**

**Supplementary Figure 11. GO term network: Sm-p80-VR1020 + rSm-p80+Resiquimod - spleen cells.**

**
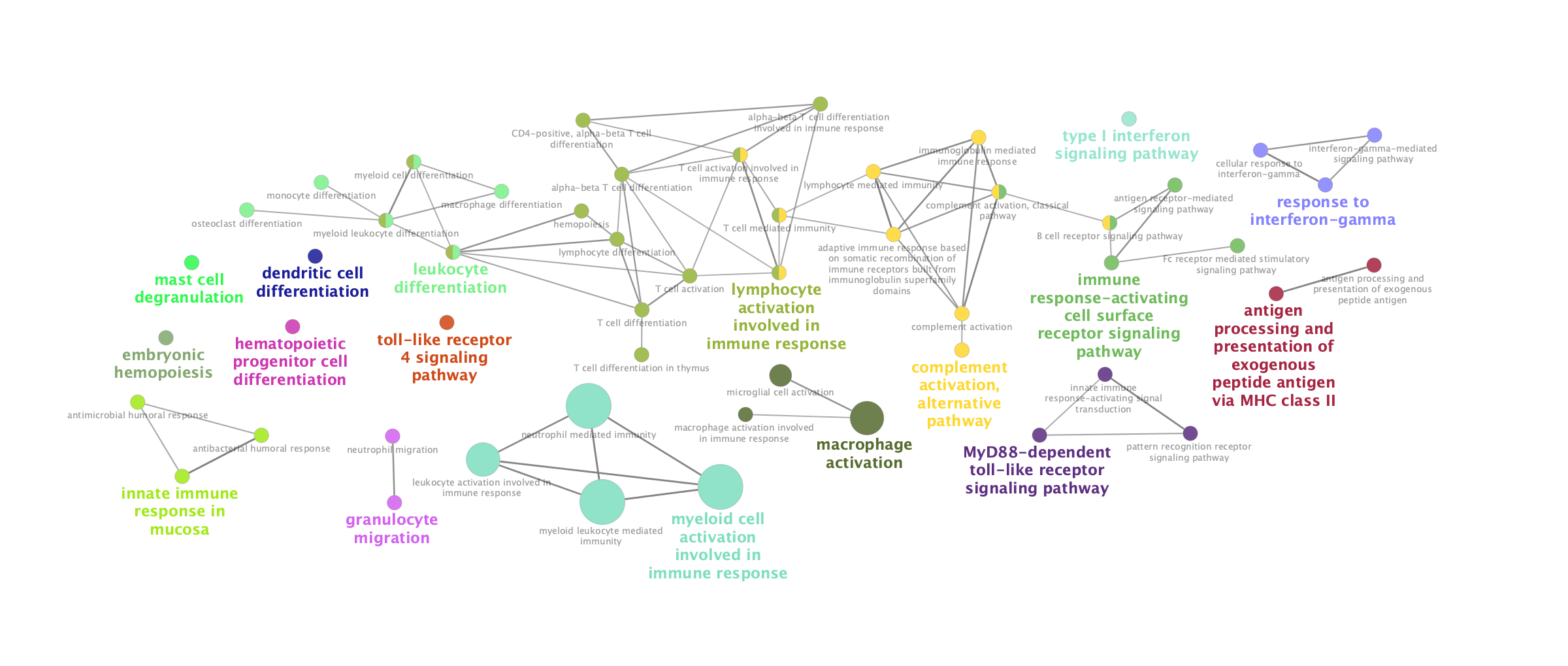
**

**Supplementary Figure 12. GO term network: Sm-p80-VR1020 + rSm-p80+Resiquimod - lymph node cells.**

**
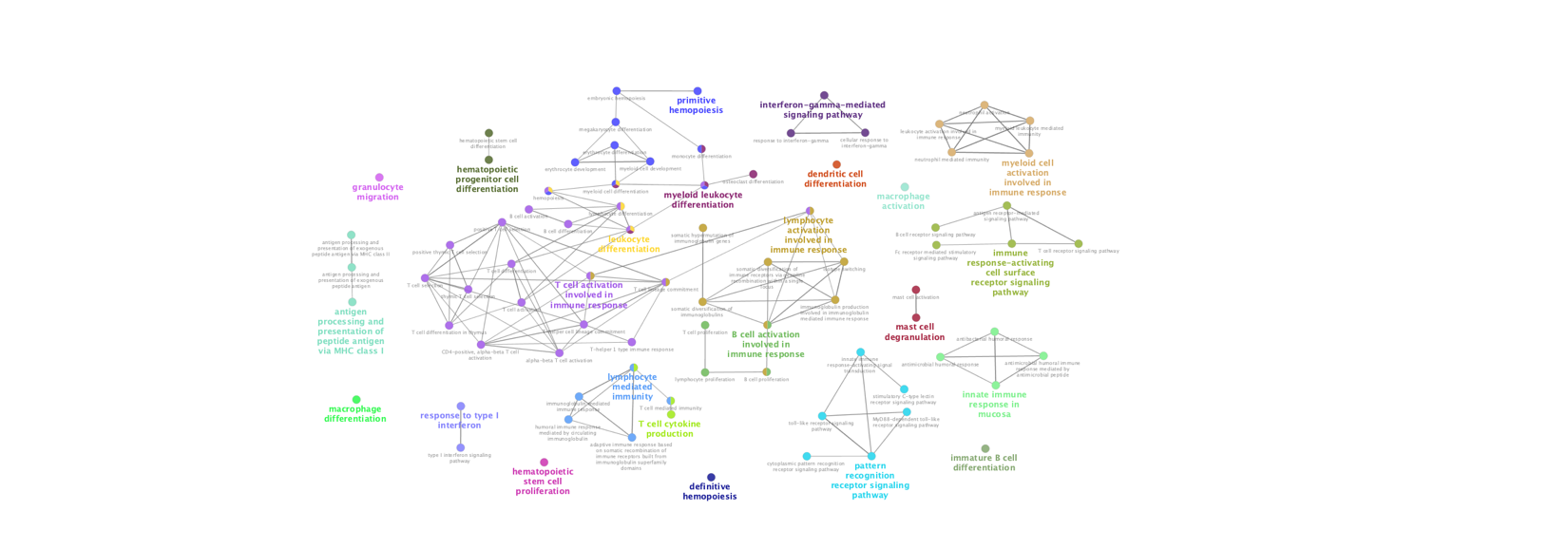
**

**Supplementary Figure 13. GO term network: rSm-p80+ODN10104 - after vaccination.**

**
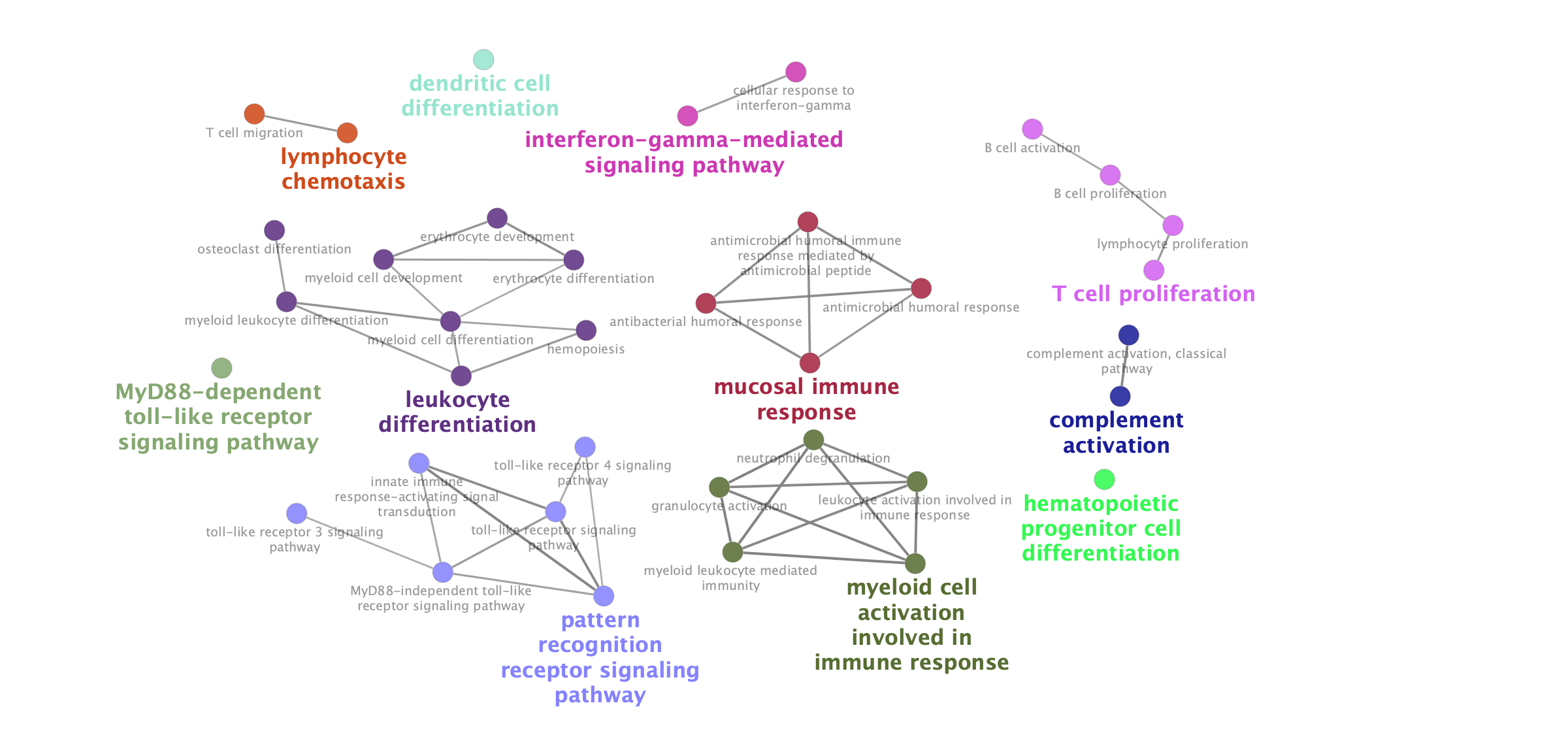
**

**Supplementary Figure 14. GO term network: rSm-p80+ODN10104 - after challenge.**

**
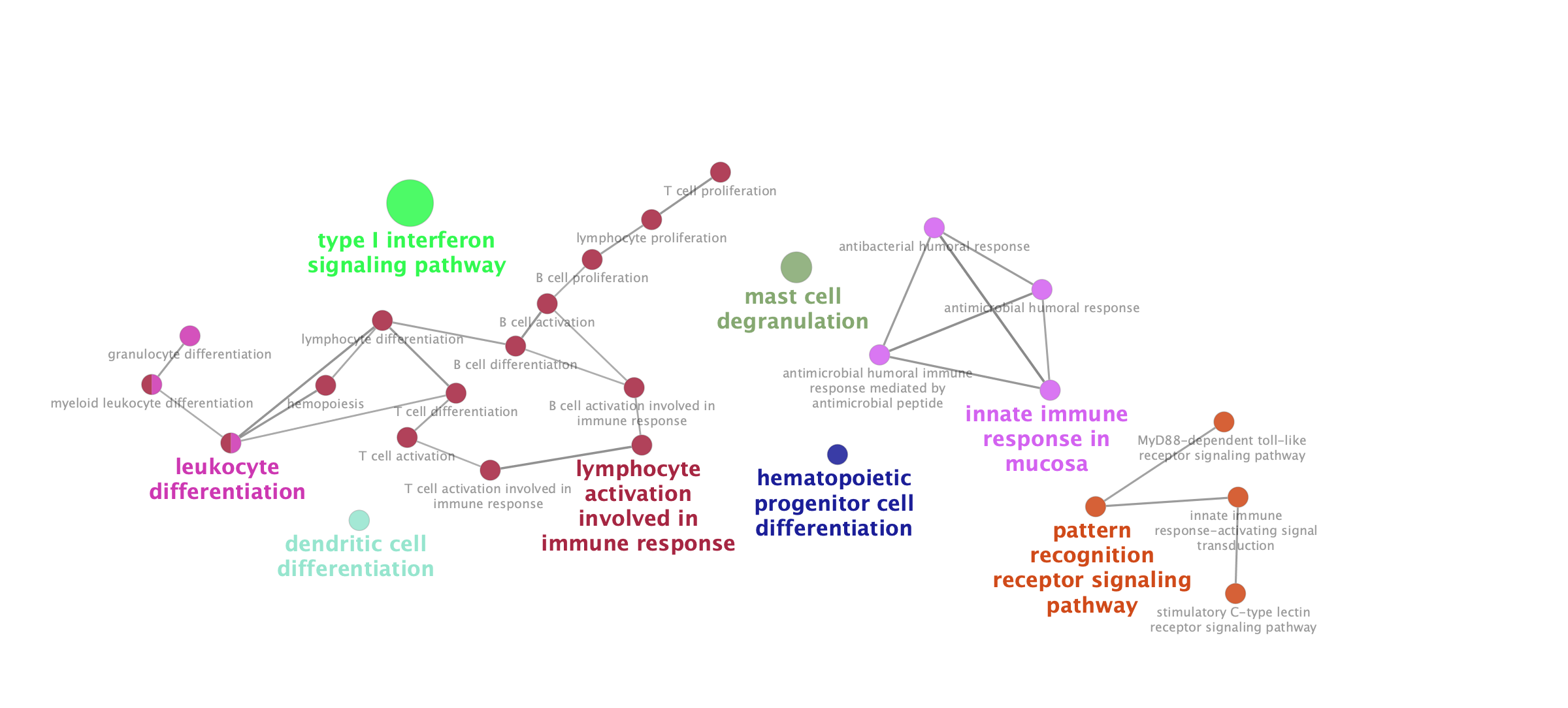
**

**Supplementary Figure 15. GO term network: rSm-p80+ODN10104 - spleen cells.**

**
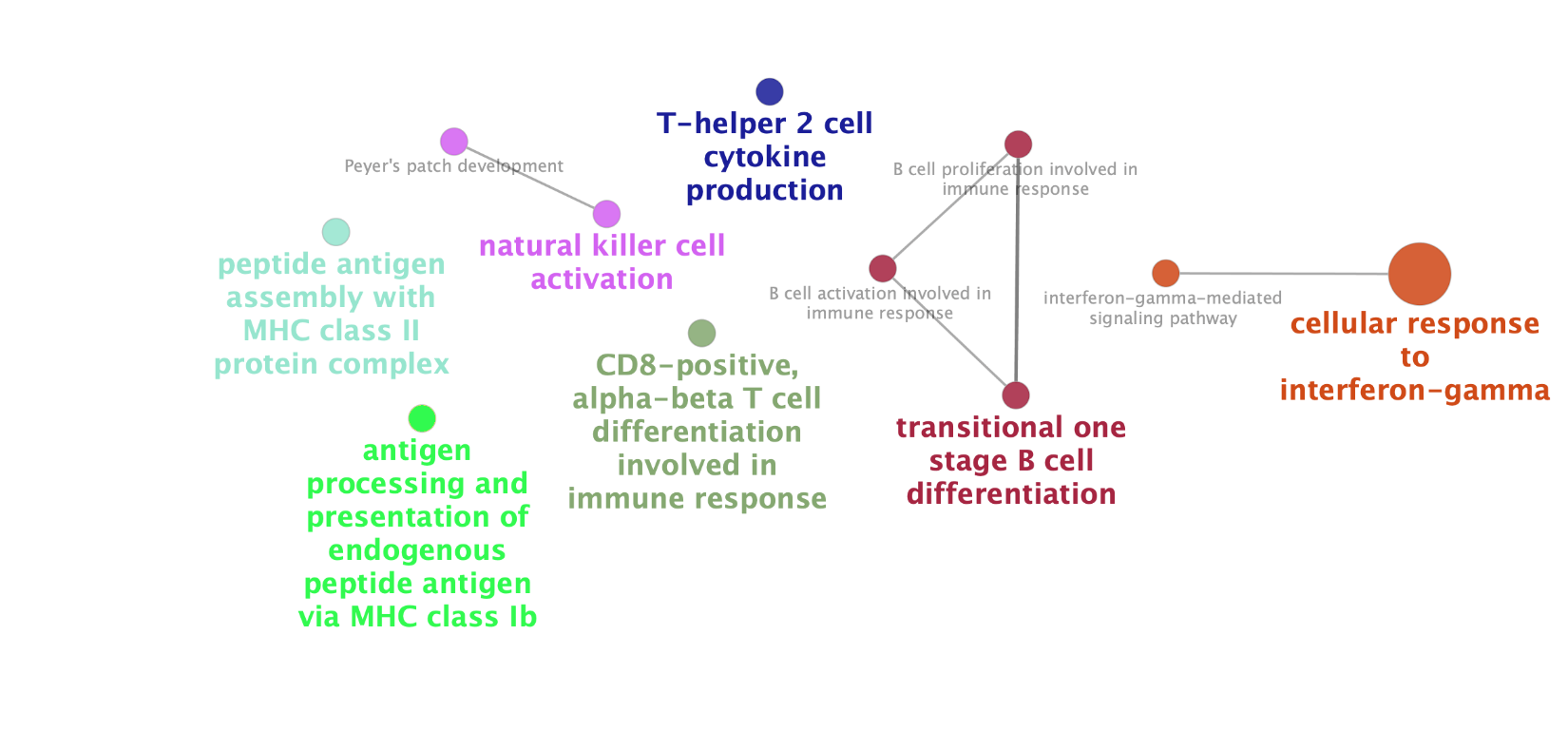
**

**Supplementary Figure 16. GO term network: rSm-p80+ODN10104 - lymph node cells.**

**
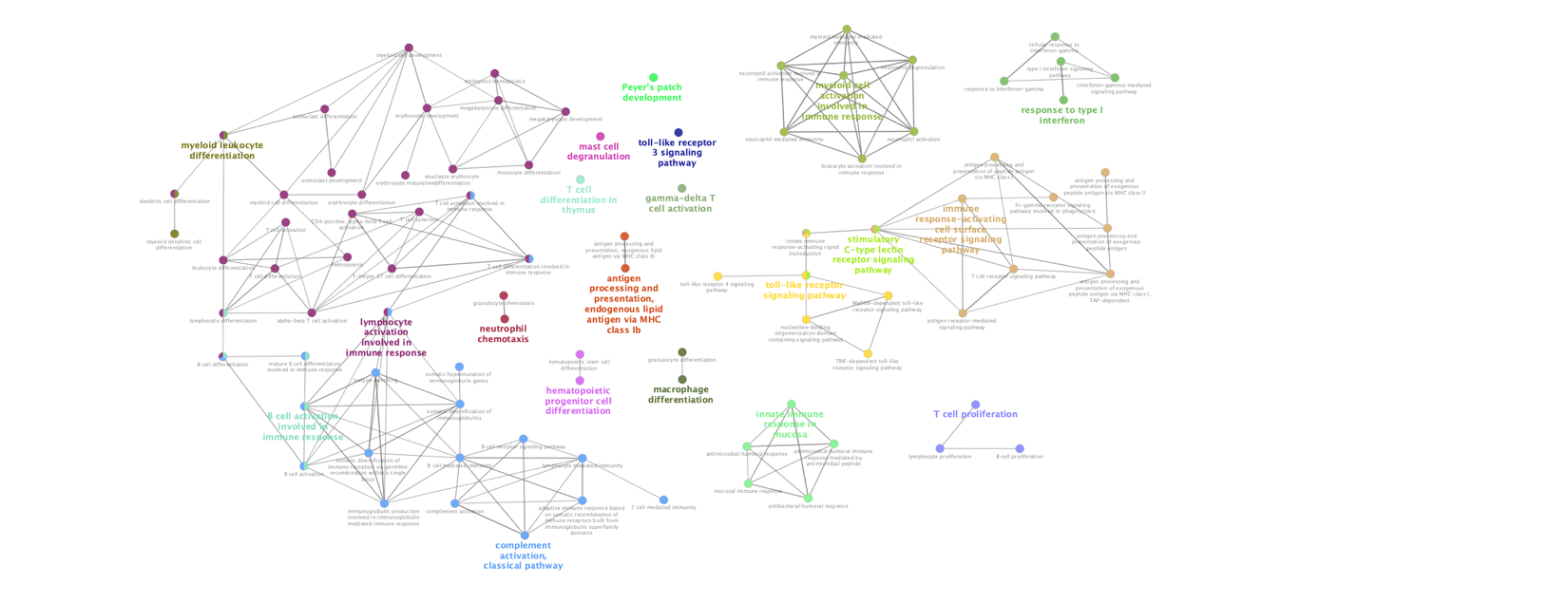
**

**Supplementary Figure 17. GO term network: rSm-p80+Resiquimod - after vaccination.**

**
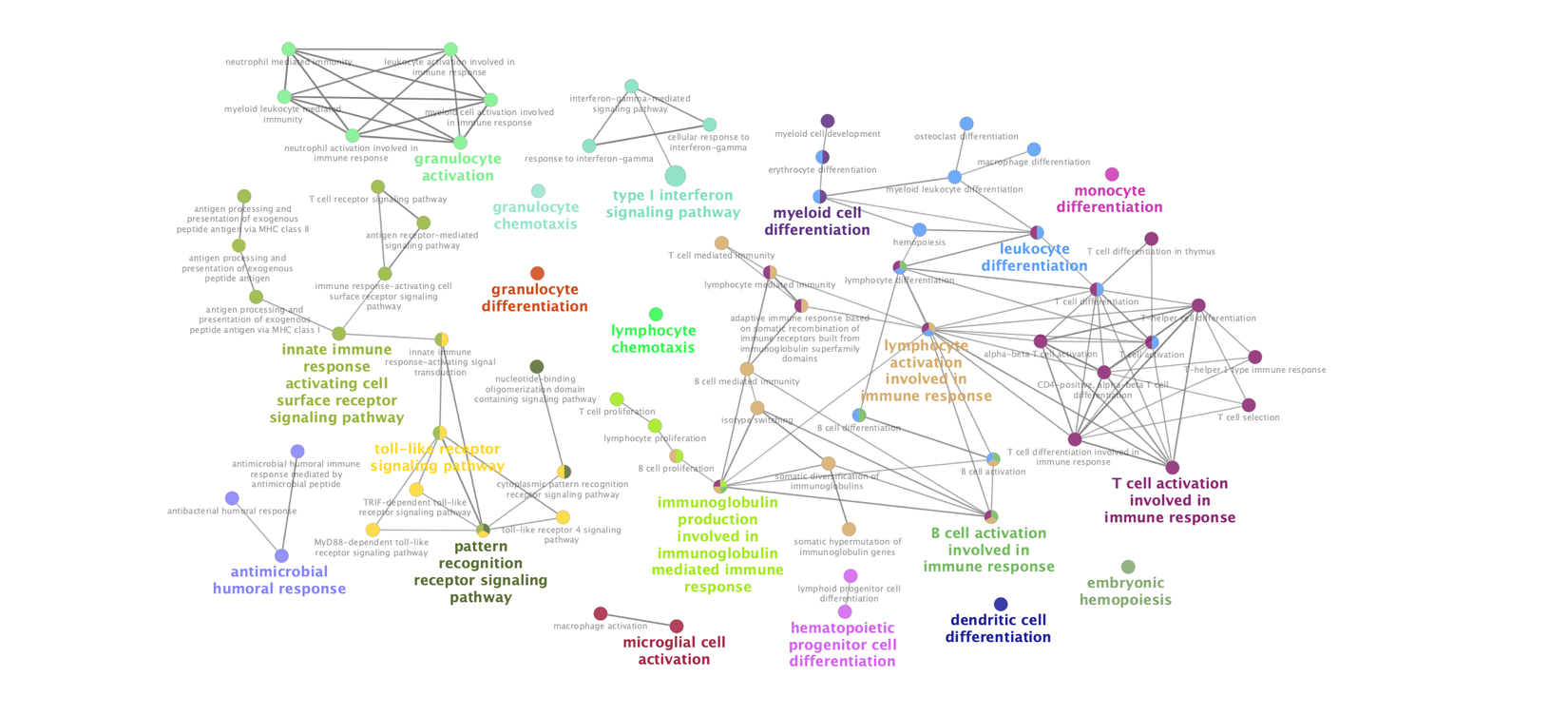
**

**Supplementary Figure 18. GO term network: rSm-p80+Resiquimod - after challenge.**

**
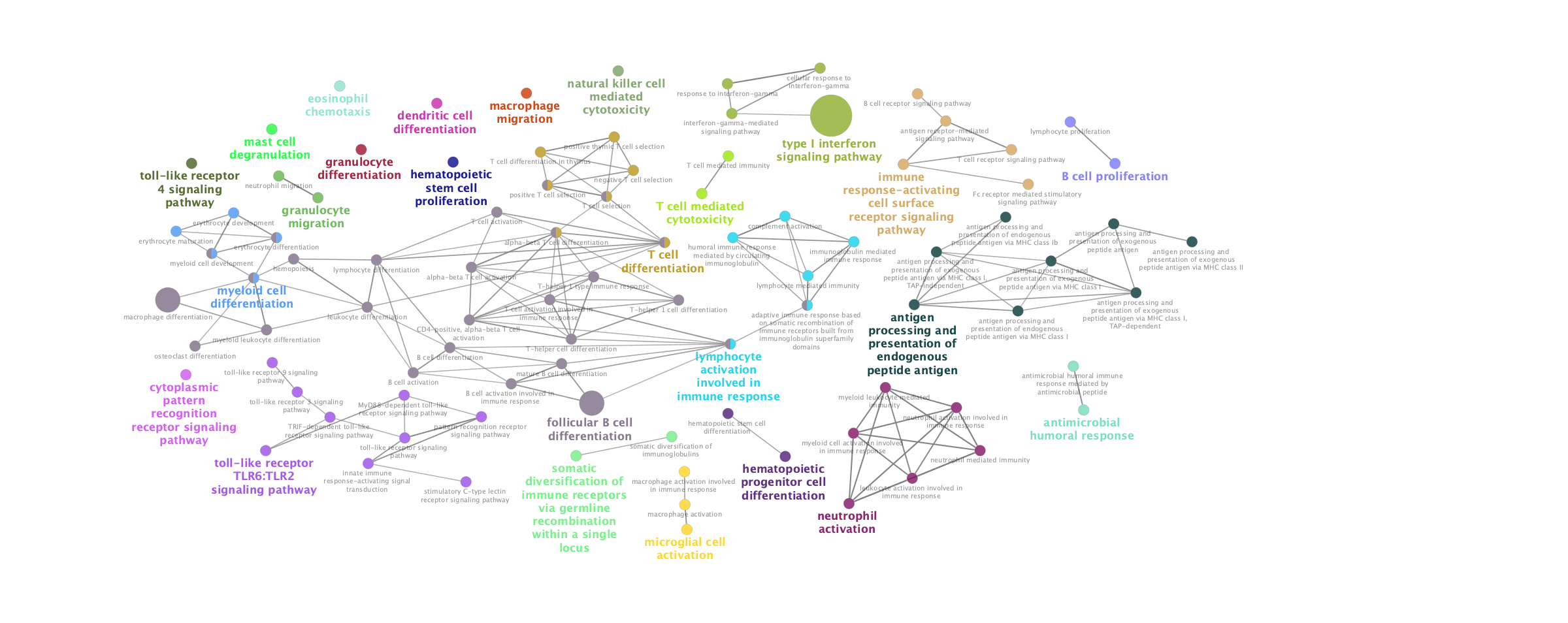
**

**Supplementary Figure 19. GO term network: rSm-p80+Resiquimod - spleen cells.**

**
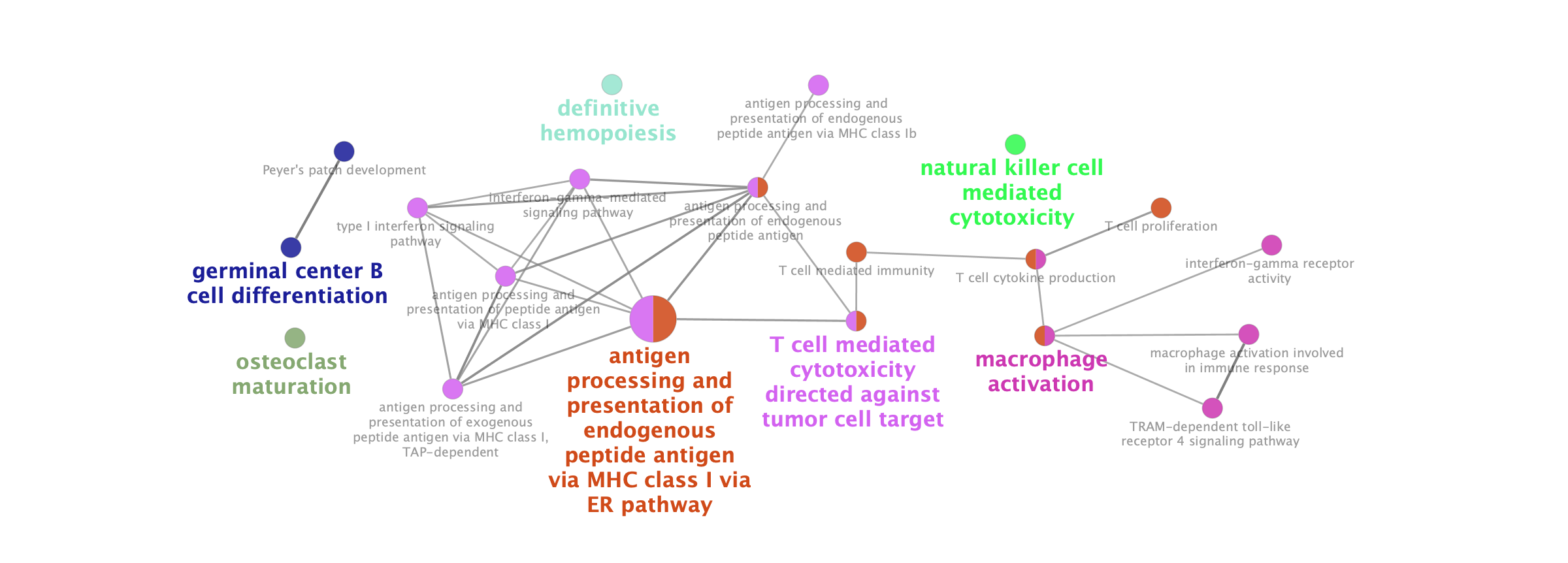
**

**Supplementary Figure 20. GO term network: rSm-p80+Resiquimod - lymph node cells.**

**
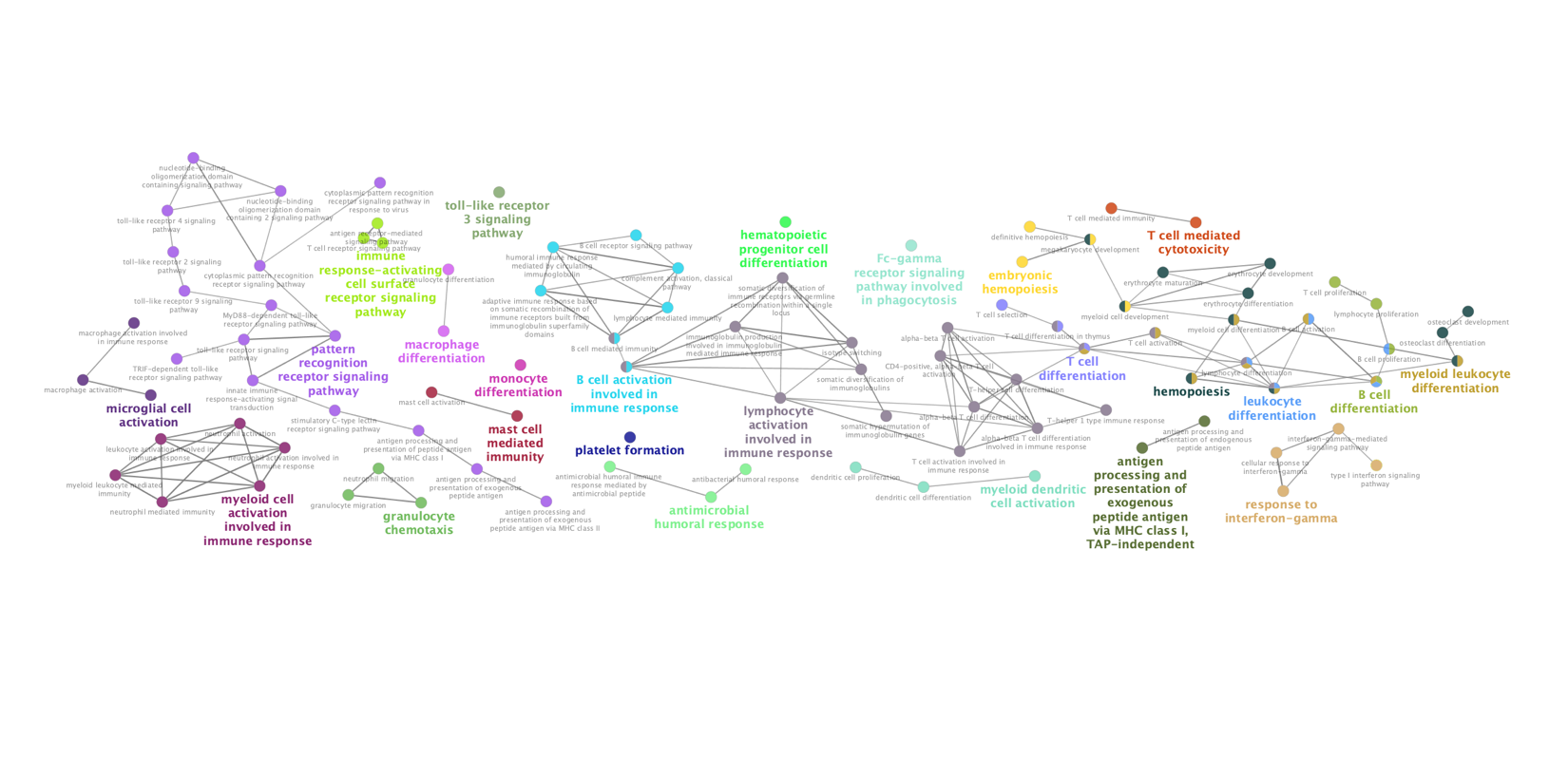
**

**Supplementary Figure 21. GO term network: rSm-p80+GLA-AF - after vaccination.**

**
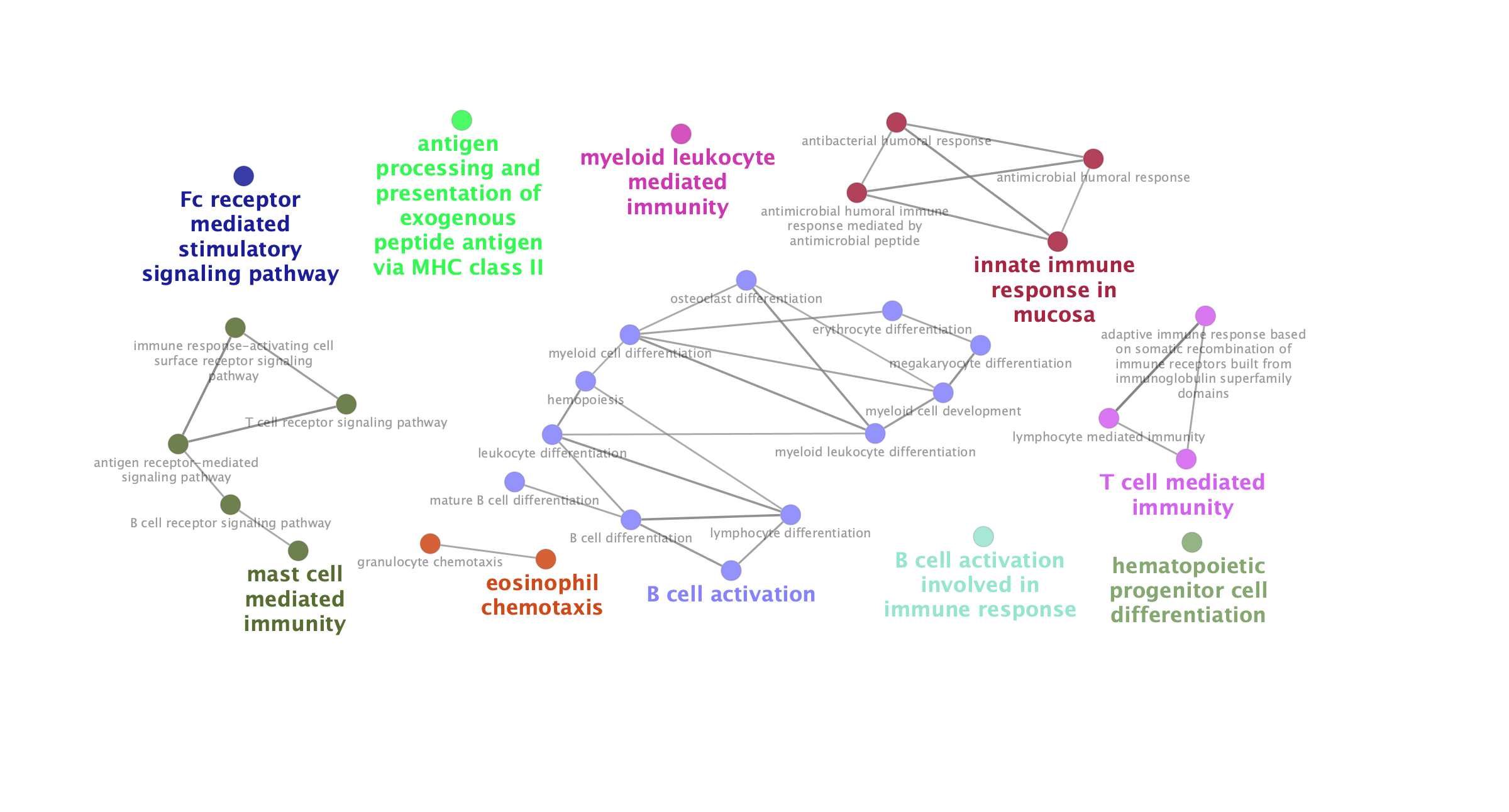
**

**Supplementary Figure 22. GO term network: rSm-p80+GLA-AF - after challenge.**

**
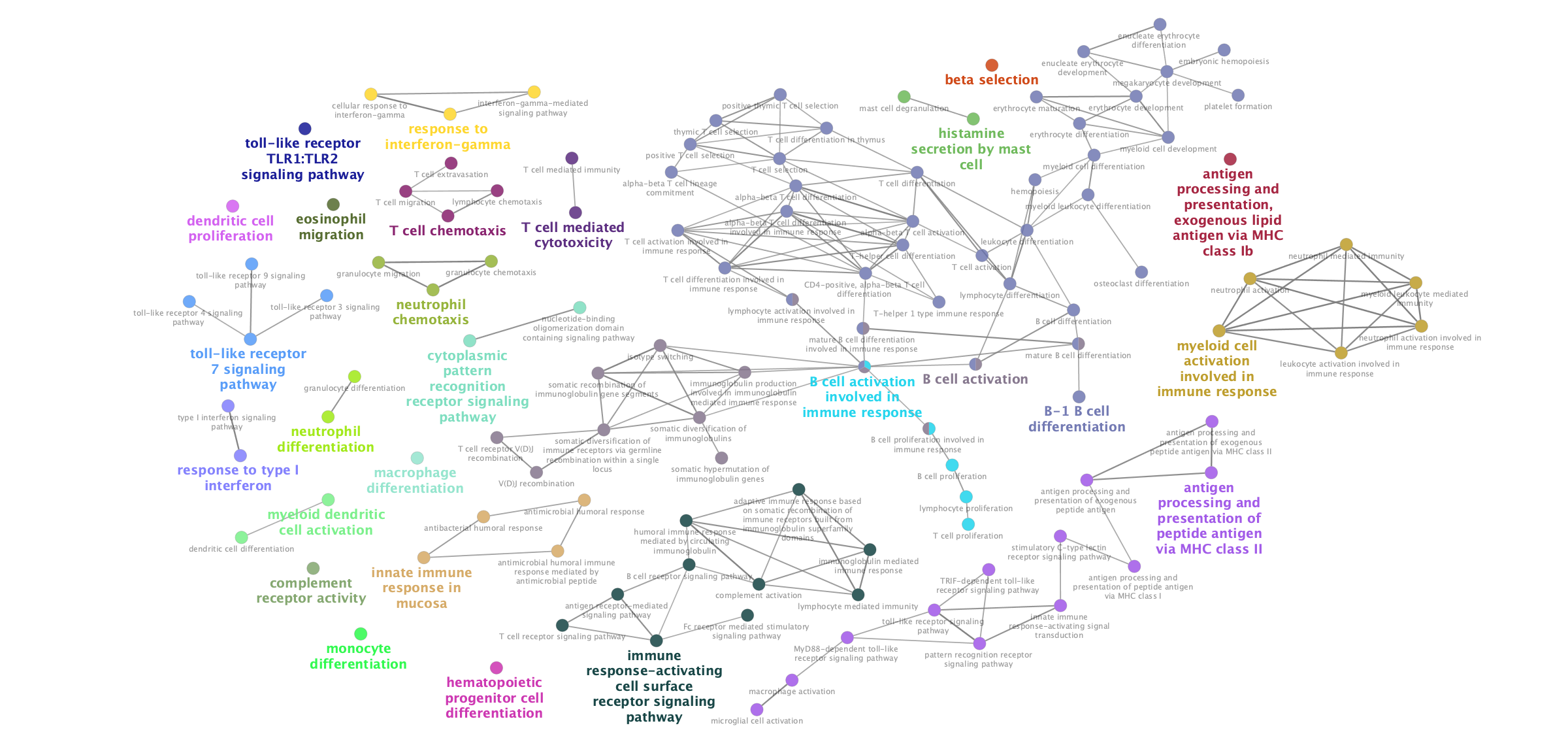
**

**Supplementary Figure 23. GO term network: rSm-p80+GLA-AF - spleen cells.**

**
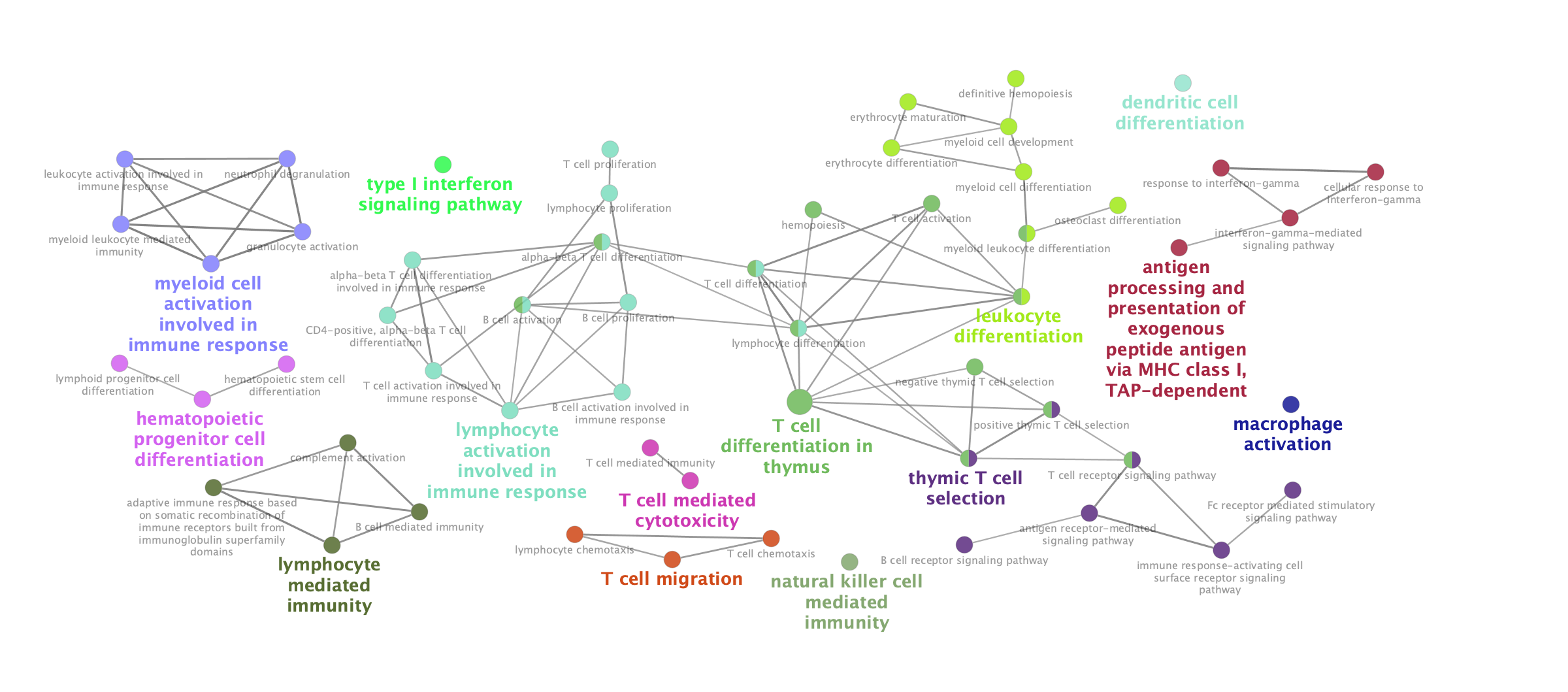
**

**Supplementary Figure 24. GO term network: rSm-p80+GLA-AF - lymph node cells.**

**
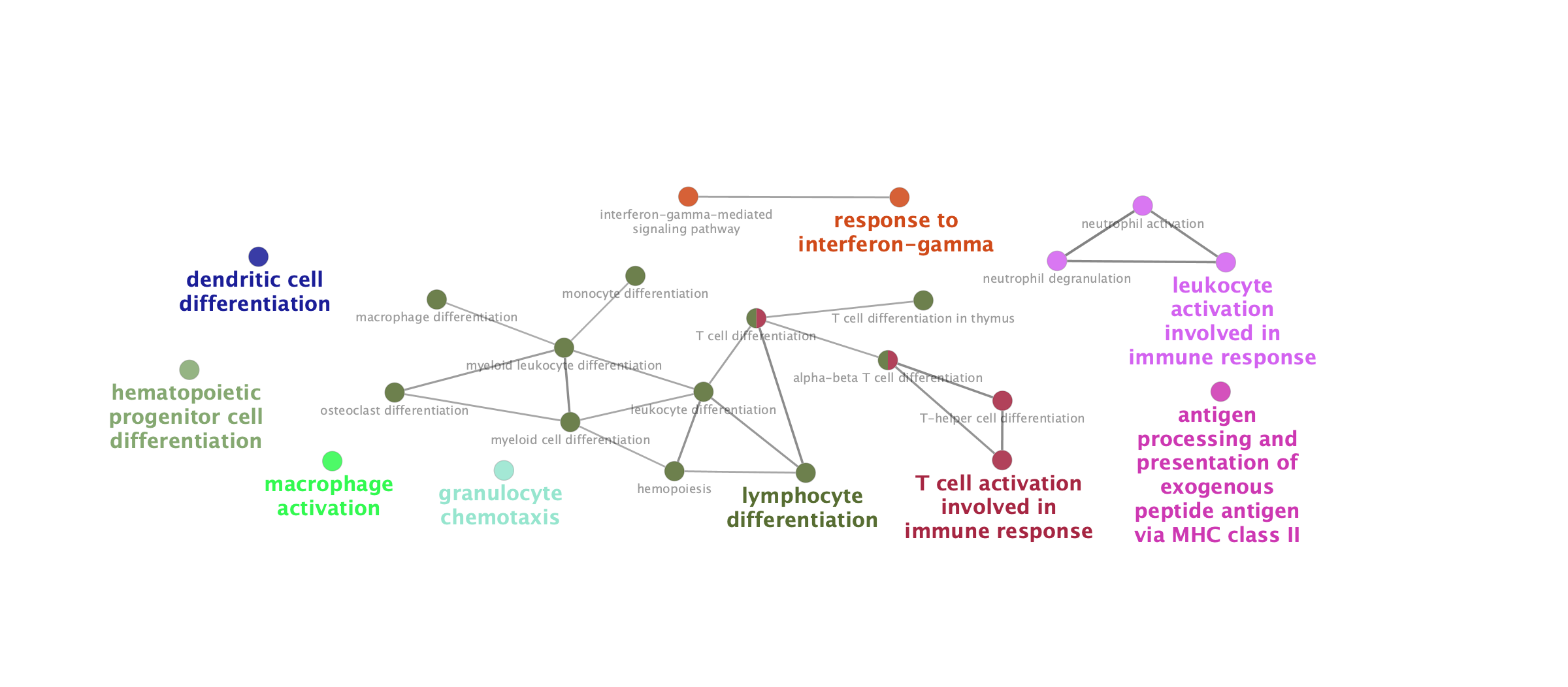
**

**Supplementary Figure 25. GO term network: rSm-p80+GLA-Alum - after vaccination.**

**
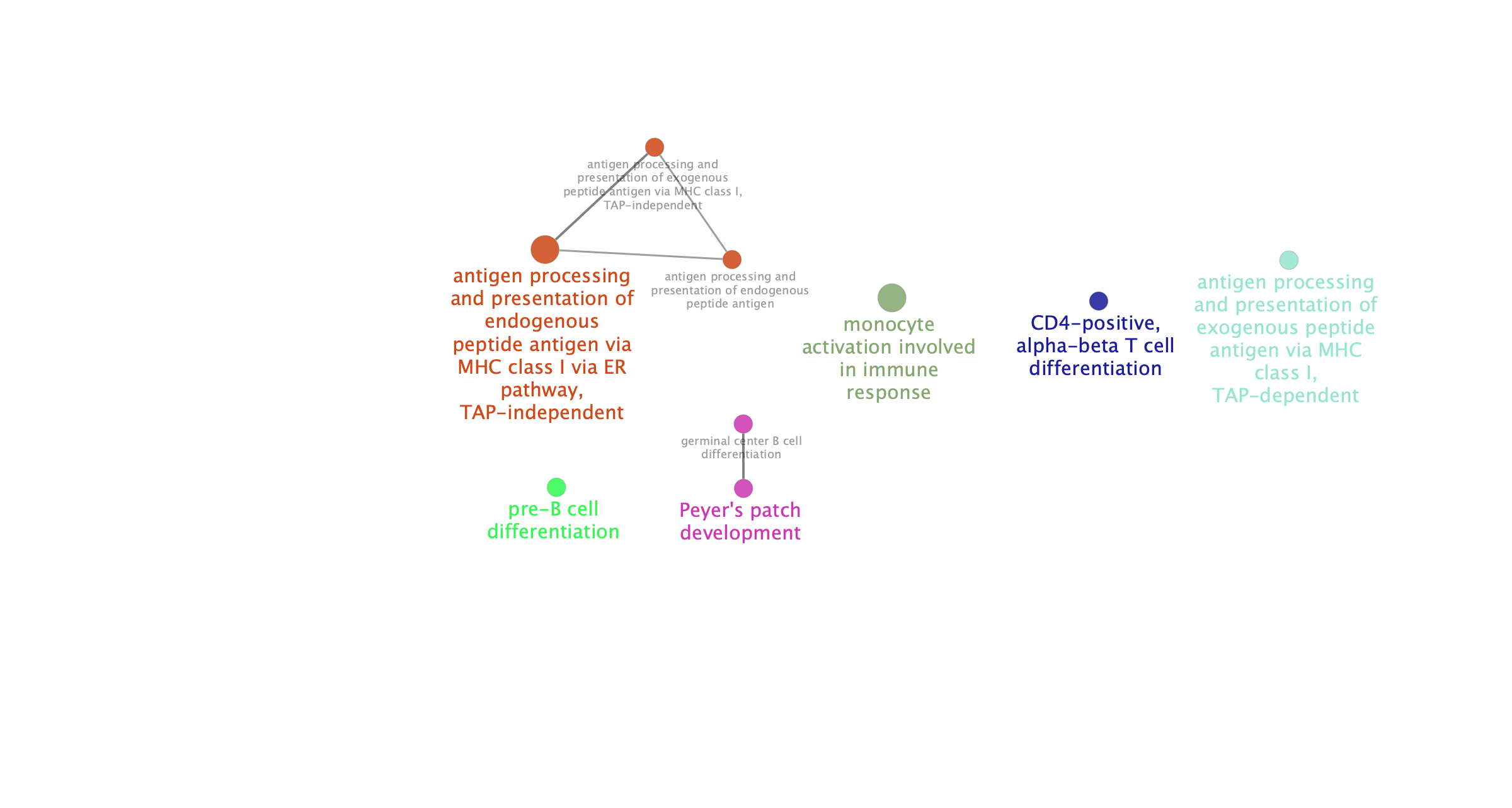
**

**Supplementary Figure 26. GO term network: rSm-p80+GLA-Alum - after challenge.**

**
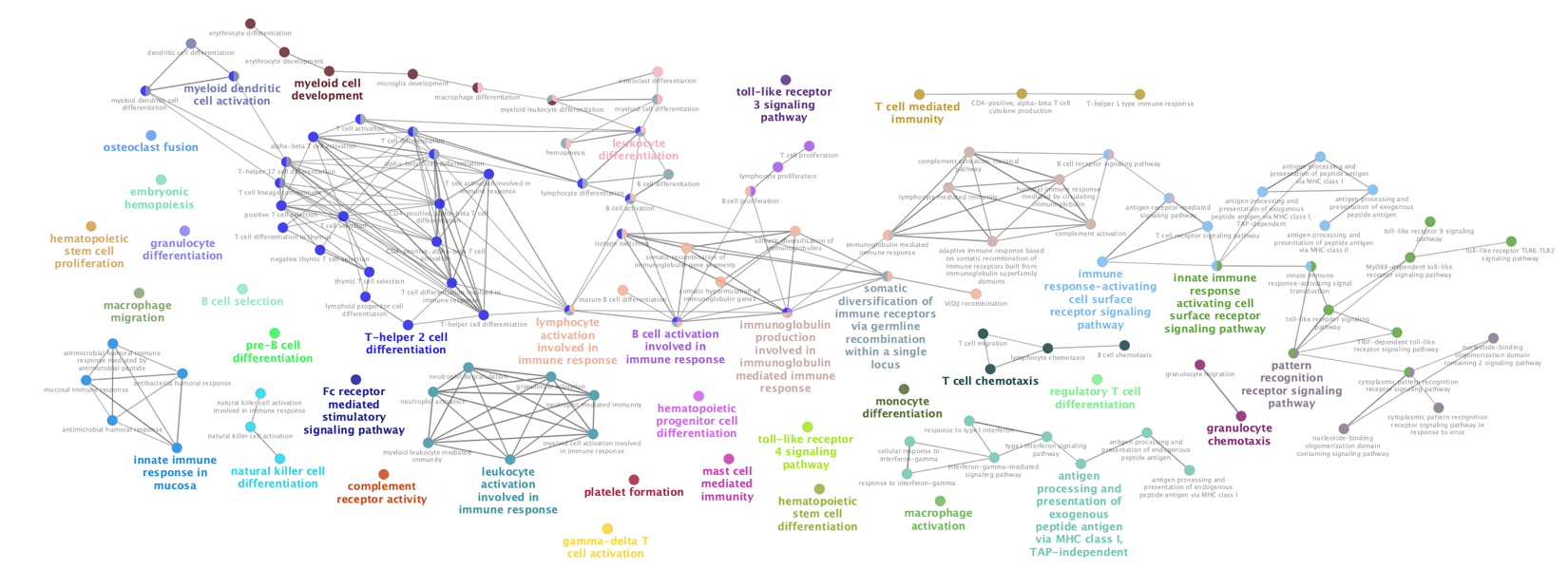
**

**Supplementary Figure 27. GO term network: rSm-p80+GLA-Alum - spleen cells.**

**
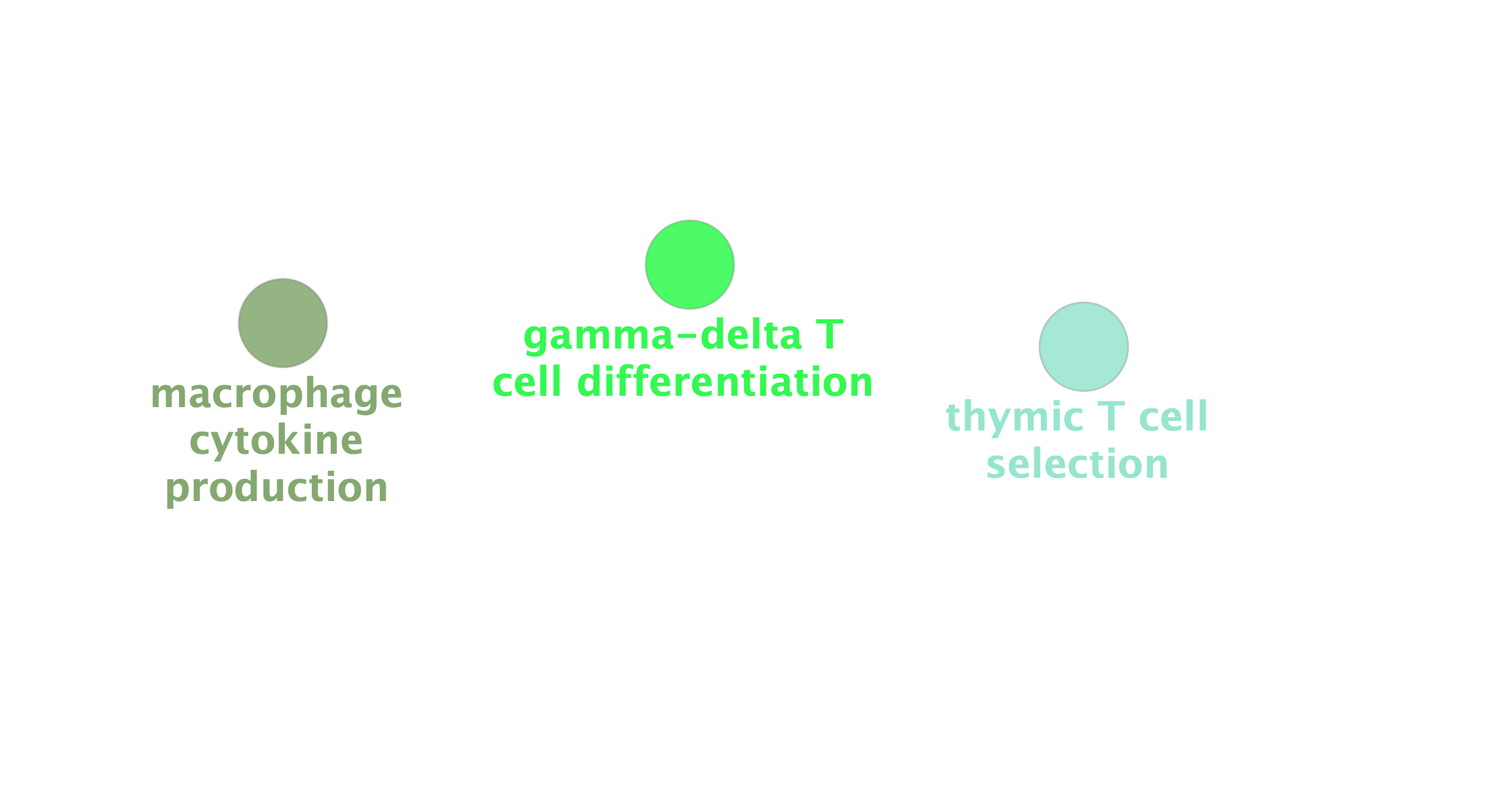
**

**Supplementary Figure 28. GO term network: rSm-p80+GLA-Alum - lymph node cells.**

**
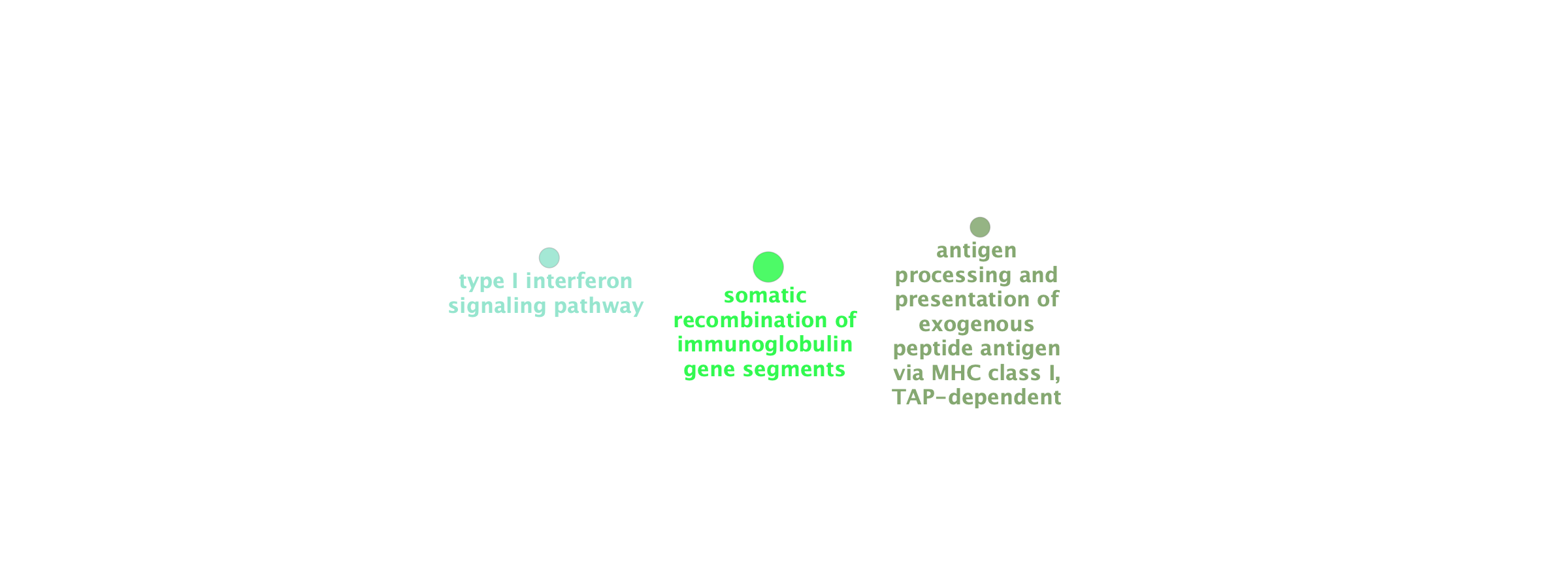
**

**Supplementary Figure 29. GO term network: rSm-p80+GLA-SE - after vaccination.**

**
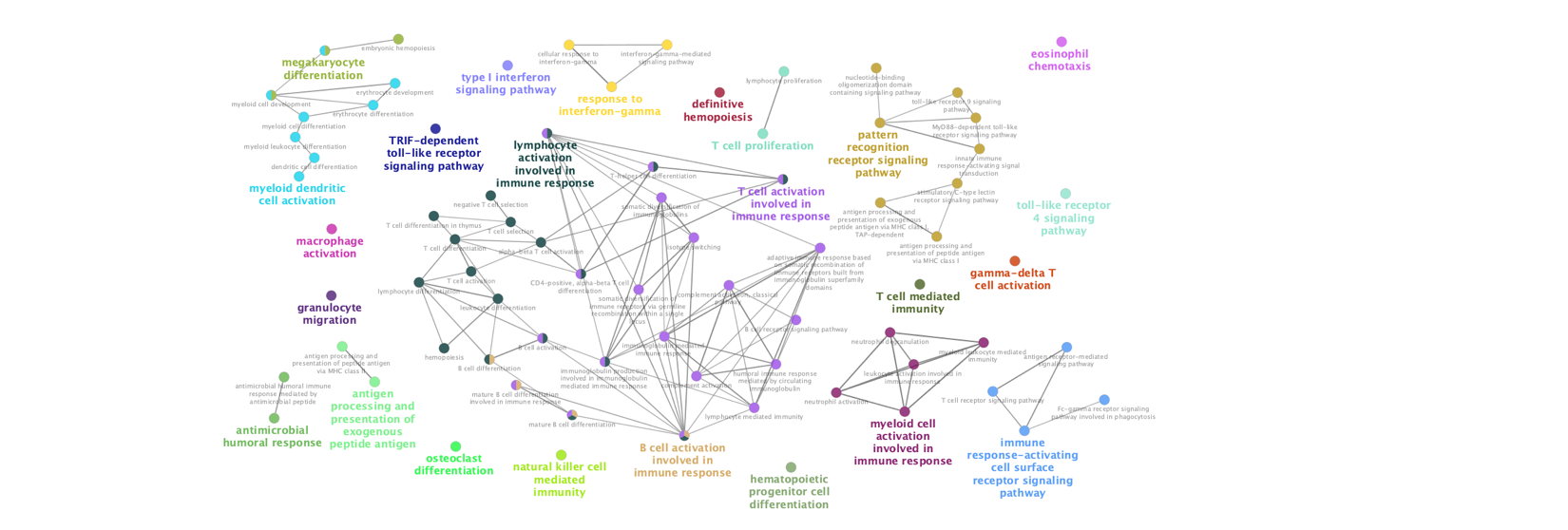
**

**Supplementary Figure 30. GO term network: rSm-p80+GLA-SE - after challenge.**

**
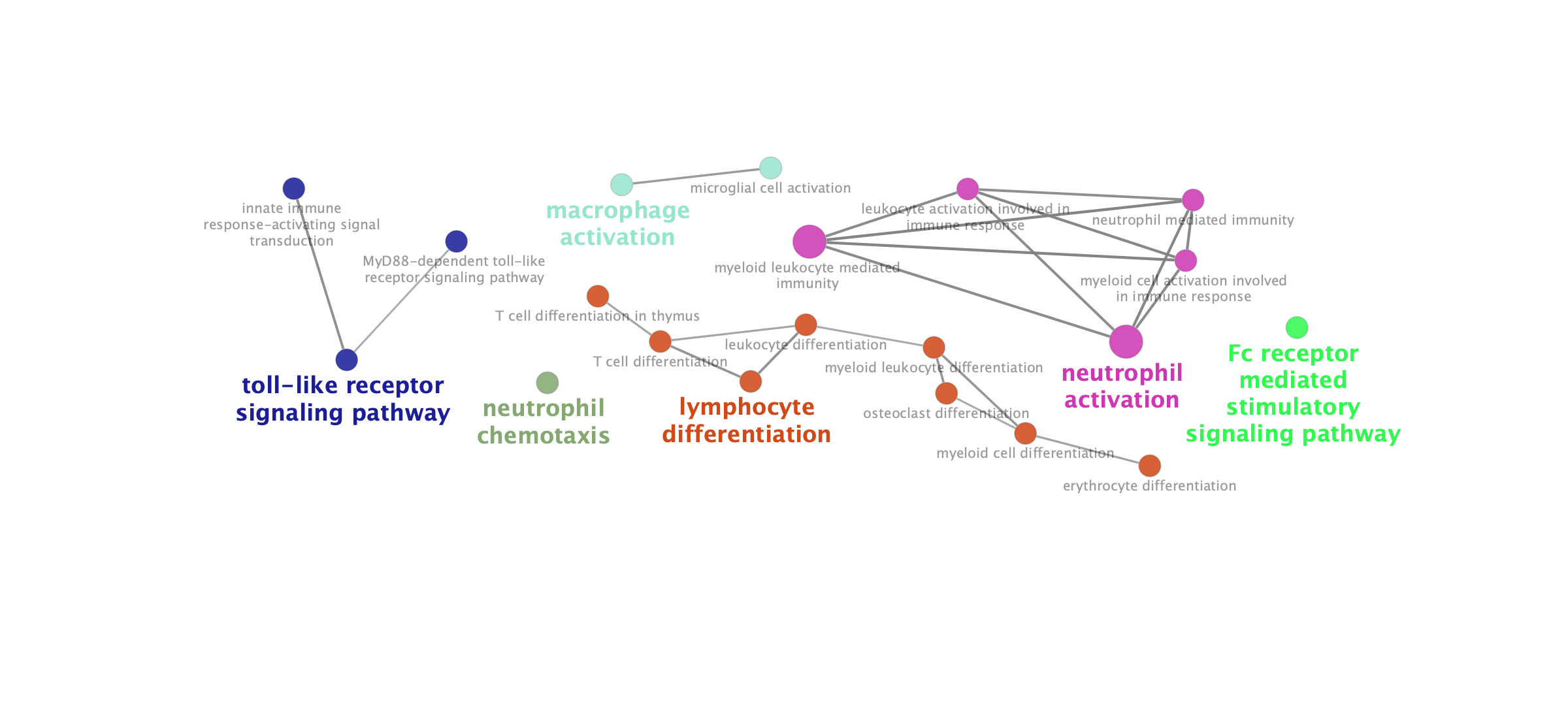
**

**Supplementary Figure 31. GO term network: rSm-p80+GLA-SE - spleen cells.**

**
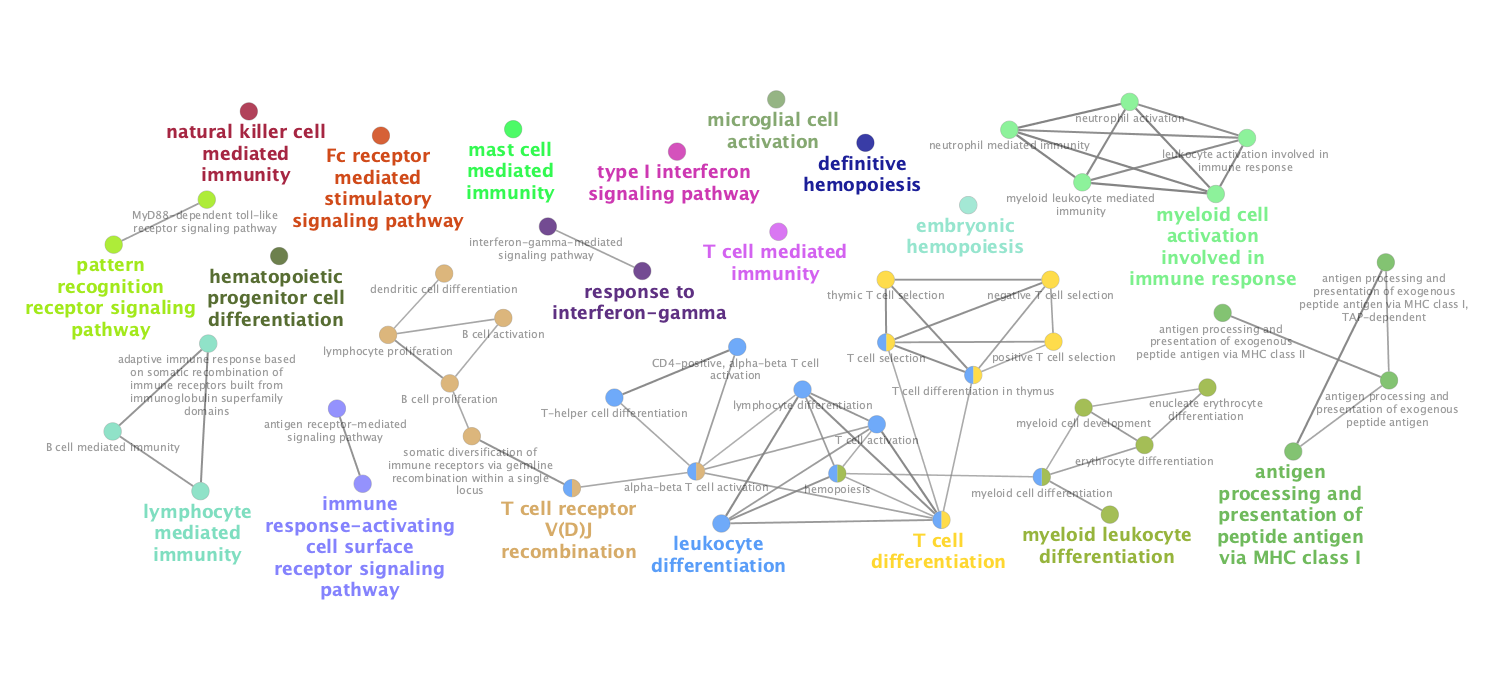
**

**Supplementary Figure 32. GO term network: rSm-p80+GLA-SE - lymph node cells.**

**
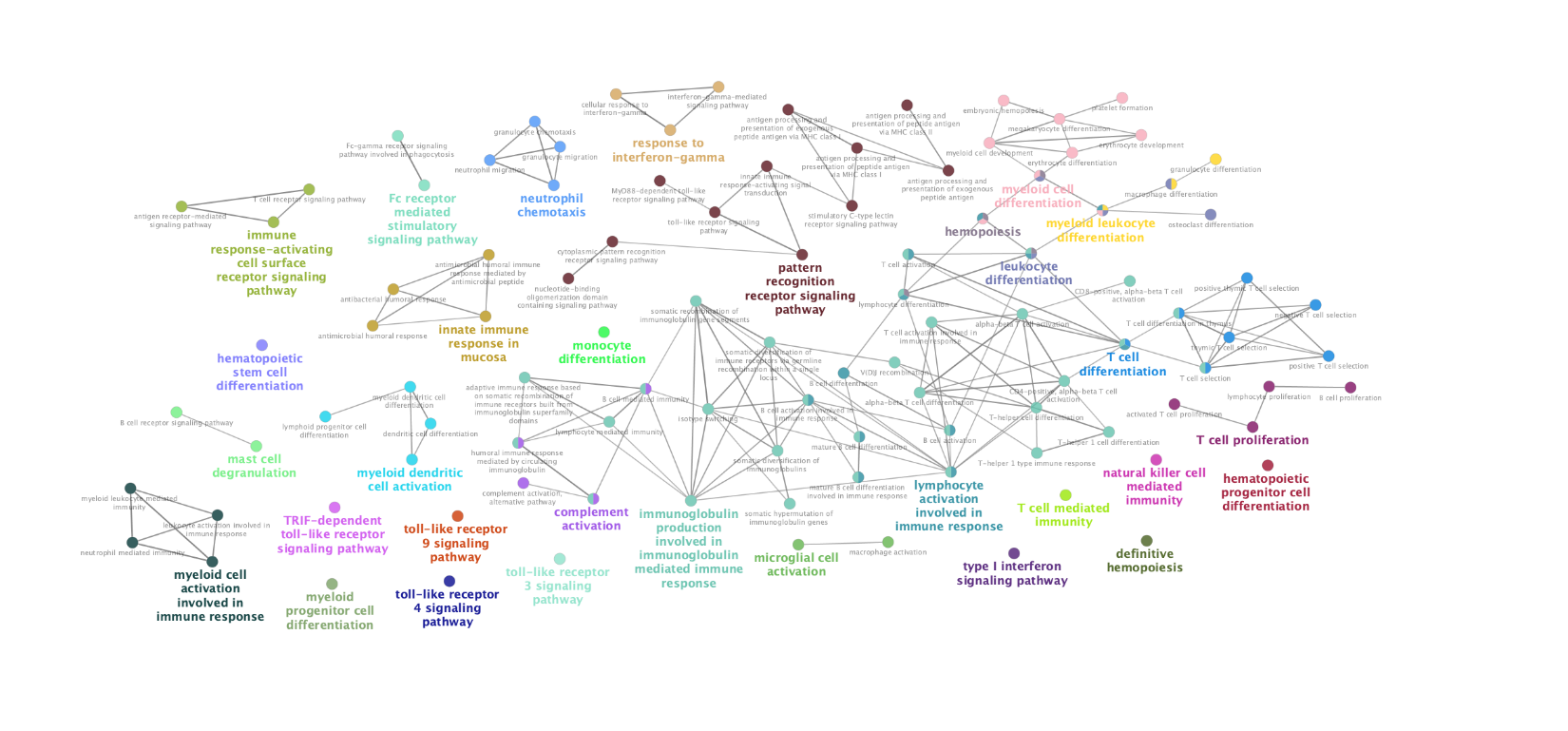
**

**Supplementary Figure 33. Number of common DEGs between strategies.** Strategy 1 denotes Sm-p80-VR1020; strategy 2 denotes Sm-p80-VR1020 + rSm-p80+ODN10104; strategy 3 denotes Sm-p80-VR1020 + rSm-p80+Resiquimod; strategy 4 denotes rSm-p80+ODN10104; strategy 5 denotes rSm-p80+Resiquimod; strategy 6 denotes rSm-p80+GLA-SE; strategy 7 denotes rSm-p80+GLA-AF; strategy 8 denotes rSm-p80+GLA-Alum.

**
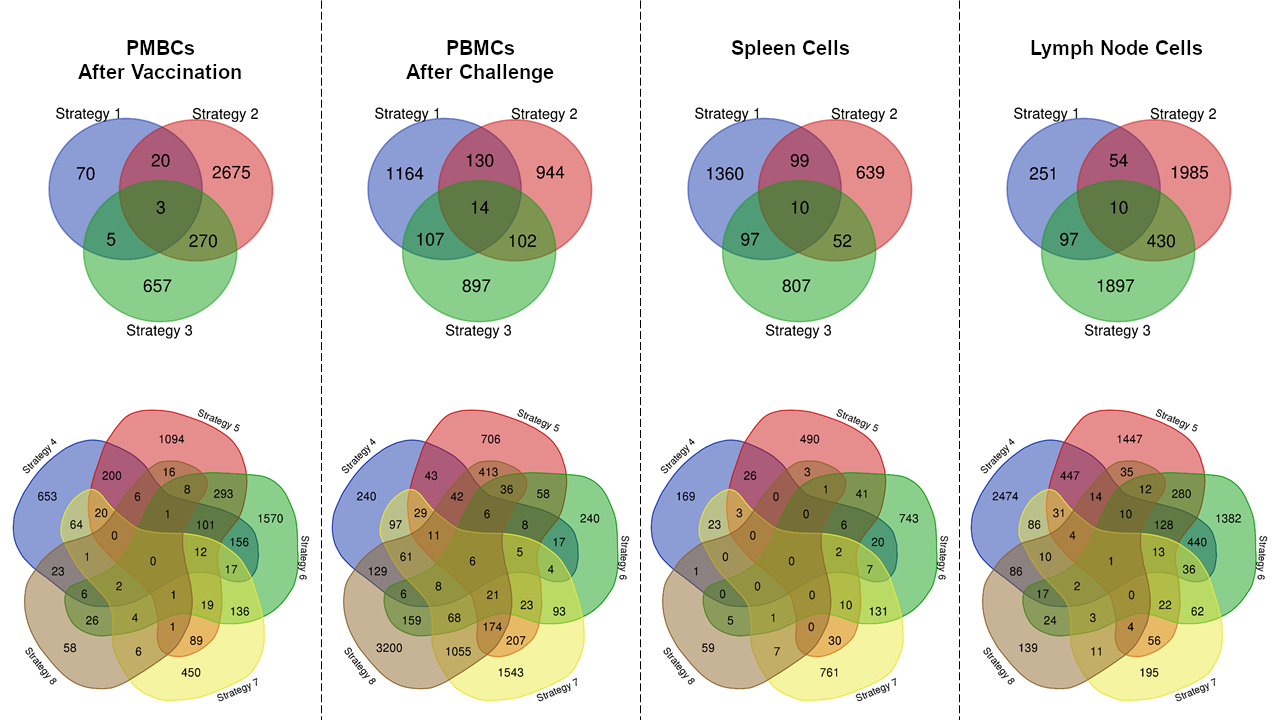
**

**Supplementary Figure 34. IgG Purification.** Representative SDS PAGE (A) and titers (B) of Sm-p80 specific IgG.

**
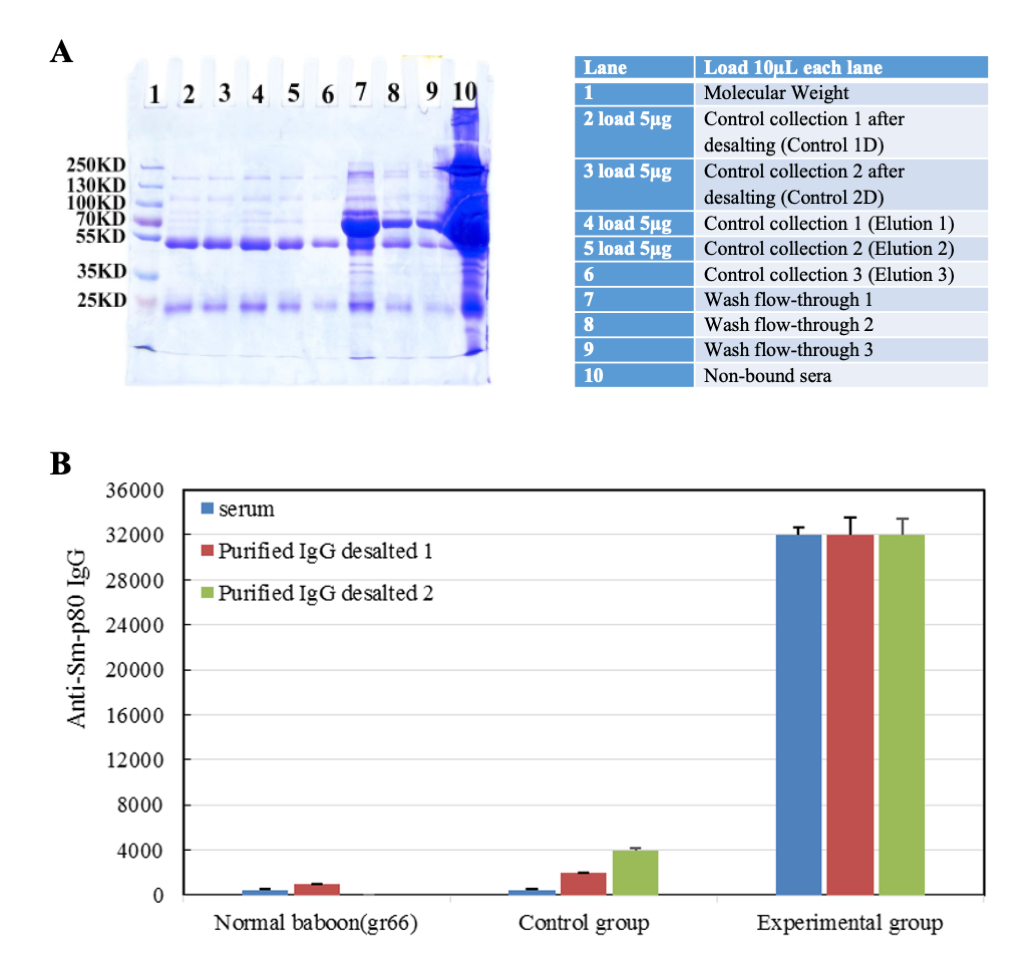
**
